# In Vitro and In Vivo Assessment of the Efficacy of Bromoageliferin, an Alkaloid Isolated from the Sponge *Agelas dilatata*, against *Pseudomonas aeruginosa*

**DOI:** 10.3390/md18060326

**Published:** 2020-06-23

**Authors:** Dawrin Pech-Puch, Mar Pérez-Povedano, Marta Martinez-Guitian, Cristina Lasarte-Monterrubio, Juan Carlos Vázquez-Ucha, Germán Bou, Jaime Rodríguez, Alejandro Beceiro, Carlos Jimenez

**Affiliations:** 1 Centro de Investigacións Científicas Avanzadas (CICA) e Departamento de Química, Facultade de Ciencias, AE CICA-INIBIC, Universidade da Coruña, 15071 A Coruña, Spain; dawrin.j.pech@udc.es (D.P.-P.); perezpovedanomaranabel@gmail.com (M.P.-P.); 2 Servicio de Microbioloxía, Instituto de Investigación Biomédica, AE CICA-INIBIC Complexo Hospitalario Universitario A Coruña, 15006 A Coruña, Spain; m.martinez.guitian@gmail.com (M.M.-G.); crlasarm@gmail.com (C.L.-M.); juan.vazquez@udc.es (J.C.V.-U.); German.Bou.Arevalo@sergas.es (G.B.)

**Keywords:** *Agelas dilatata*, Yucatan Peninsula, pyrrole-imidazole alkaloids, *Pseudomonas aeruginosa*, structure-activity relationships, antibacterial, biofilm inhibition, *Galleria mellonella*

## Abstract

The pyrrole-imidazoles, a group of alkaloids commonly found in marine sponges belonging to the genus *Agelas*, display a wide range of biological activities. Herein, we report the first chemical study of the secondary metabolites of the sponge *A. dilatata* from the coastal area of the Yucatan Peninsula (Mexico). In this study, we isolated eight known alkaloids from an organic extract of the sponge. We used NMR and MS analysis and comparison with existing databases to characterize the alkaloids: ageliferin (**1**), bromoageliferin (**2**), dibromoageliferin (**3**), sceptrin (**4**), nakamuric acid (**5**), 4-bromo-1H-pyrrole-2-carboxylic acid (**6**), 4,5-dibromopyrrole-2-carboxylic acid (**7**) and 3,7-dimethylisoguanine (**8**). We also evaluated, for the first time, the activity of these alkaloids against the most problematic multidrug-resistant (MDR) pathogens, i.e., the Gram-negative bacteria *Pseudomonas aeruginosa*, *Klebsiella pneumoniae* and *Acinetobacter baumannii*. Bromoageliferin (**2**) displayed significant activity against *P. aeruginosa*. Comparison of the antibacterial activity of ageliferins **1**–**3** (of similar structure) against *P. aeruginosa* revealed some relationship between structure and activity. Furthermore, in in vitro assays, **2** inhibited growth and biofilm production in clinical strains of *P. aeruginosa*. Moreover, **2** increased the survival time in an in vivo *Galleria mellonella* model of infection. The findings confirm bromoageliferin (**2**) as a potential lead for designing new antibacterial drugs.

## 1. Introduction

Multidrug-resistant bacterial infections represent a serious global health problem [1], causing an estimated 700,000 deaths a year worldwide. If this rising trend in antibiotic resistance is not reversed in the coming years, it could lead to 10 million people dying every year and the economic impact of approximately 1% reduction of the world’s gross domestic product (GDP) and there would be a 5–7% loss in developing countries by 2050 [2]. The increase in bacterial resistance (especially in relation to Gram-negative bacteria), together with the scarce development of new antimicrobial compounds in the last few decades, has led to the current situation in which very few or even no antibiotics are available to treat complicated infections [3]. The need for new therapeutic options to treat multidrug resistant pathogen infections is therefore indisputable. 

Biofilm formation is an important bacterial survival strategy. A biofilm is a protective extracellular matrix which enables bacteria to resist the action of antibiotics and the host immune response. Biofilm production is a major virulence factor in infections such as periodontitis, native valve endocarditis and cystic fibrosis [4]. The development of new molecules should therefore be directed towards biofilm-specific targets in order to increase the therapeutic arsenal available to treat multidrug-resistant pathogens.

The traditional approach to identifying new antibacterial therapies is to search for bacteriostatic or bactericidal compounds from natural sources and synthetic pathways. Natural products have been one of the most prolific sources of new leads in modern drug discovery [5]. The marine environment, a rich source of chemically diverse, biologically active natural products, serves as an invaluable resource in the ongoing search for novel antimicrobial compounds [6]. 

Sponges belonging to the genus *Agelas* constitute an important source of marine pyrrole-imidazole alkaloids [7,8]. Since the structural elucidation of the first alkaloid, dibromophakellin, almost 50 years ago [9], more than 200 analogues isolated from a variety of tropical sponges have been found to display a wide range of biological properties including antitumor, antifungal and antibacterial activities [10,11].

In this study, we continued to search for natural bioactive products from marine organisms [12,13] more specifically from those collected off the coast of the Yucatan Peninsula (Mexico) [14,15]. We focused our attention on the sponge *A. dilatata* collected from Cozumel Island, because of the antibacterial activity detected in organic extracts of the sponge and the lack of reported chemical studies of its secondary metabolites. The only previously published studies concerning *A. dilatata* are a comparative study of the microbial diversity and analysis of the fatty acid composition of specimens collected in the Bahamas [16] and a taxonomic study of specimens of the genus *Agelas* from the Caribbean Sea [17]. The present study is the first chemical assessment of the natural products isolated from organic extracts of *A. dilatata*. The study involved the isolation and structural characterization of eight known alkaloids, most of which belong to the family of bromopyrrole-imidazole alkaloids. Furthermore, with the aim of obtaining further insights into the antibiotic activity of these compounds, we evaluated the minimum inhibitory concentrations (MICs) of the three pathogenic species classified by the WHO as critical in regard to the need for new antimicrobial therapies: *Pseudomonas aeruginosa*, *Klebsiella pneumoniae* and *Acinetobacter baumannii*. These species over the last decades have become a major public health crisis worldwide, because are responsible for a large number of hospital-acquired and nosocomial infections and by their increasing development of antimicrobial resistance [18]. In addition, the antimicrobial efficacy of these alkaloids was assessed by measuring the anti-biofilm activity and survival time in an in vivo *Galleria mellonella* infection model. 

## 2. Results and Discussion

### 2.1. Extraction, Isolation and Structural Elucidation

**Figure 1:**
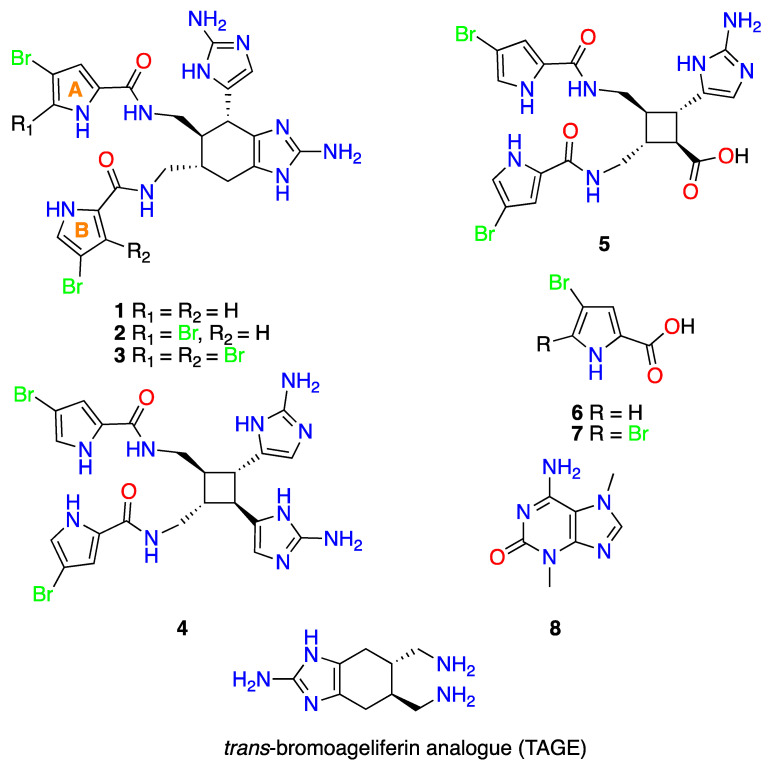
Stuctures of alkaloids **1**–**8**, isolated from the sponge *A. dilatata*, and of TAGE.

Specimens of the sponge *A. dilatata*, collected from Cozumel Island, state of Quintana Roo, Yucatan Peninsula (Mexico), were extracted several times with a 1:1 mixture of CH_3_OH/CH_2_Cl_2_ to yield an organic extract which displayed antibacterial activity in a bioassay against three Gram-negative pathogens. The extract was then partitioned between water and solvents of increasing polarity to produce hexane, *n*-butanol, dichloromethane and a final aqueous methanolic fraction. Bioassay-guided fractionation allowed us to select the *n*-butanol and aqueous methanolic bioactive fractions enriched with bromopyrrole alkaloids from the oroidin family. The core skeleton of these compounds was deduced by MS analysis, which revealed the typical bromine isotopic peak clusters, and by ^13^C NMR studies, which showed the characteristic carbon chemical shifts at *δ*_C_ ~160 (CO), ~125 (C), ~124 (CH), ~118 (CH) and ~98 (C-Br) [19]. The *n*-BuOH fraction was subjected to solid phase extraction (SPE)-C18 (H_2_O/CH_3_OH/CH_2_Cl_2_ gradient system), and the resulting fractions were separated by reversed-phase HPLC to yield compounds **1**, **2** and **4**–**8**. In addition, the aqueous methanolic fraction was subjected to reversed-phase HPLC, yielding compounds **1**–**3** and **5**–**7** (Figure 1).

Comparison of the NMR spectra (1D and 2D NMR) and MS data for **1**–**8** with previously reported data allowed us to determine the structures of the compounds (See Figure 1 and Figures S1–S24 in Suplementary Material. We were thus able to identify five oroidin derivatives, three of which were the ageliferins resulting from putative 4π + 2π (Diels-Alder) cyclization [20,21,22]: ageliferin (**1**), bromoageliferin (**2**), and dibromoageliferin (**3**); another two resulting from putative 2π + 2π cyclization: sceptrin (**4**) [23] and nakamuric acid (**5**) [24]; two pyrrole derivatives: 4-bromo-1H-pyrrole-2-carboxylic acid (**6**) [25] and 4,5-dibromopyrrole-2-carboxylic acid (**7**) [26], and finally, the nitrogenated base 3,7-dimethylisoguanine (**8**) [27]. Although **1**–**8** have been isolated from other sponges in the genus *Agelas*, this is first time that they have been isolated from *A. dilatata*. Once the chemical structures were determined, the alkaloids were subjected to several biological studies. 

### 2.2. Antimicrobial Susceptibility Testing 

Minimum inhibitory concentration (MIC) assays were performed with all of the alkaloids (**1**–**8**) against reference bacterial strains and clinical isolates (see Table 1). Four categories of antibacterial activity were established: high activity (MIC ≤ 8 mg/L); moderate activity (MIC = 16–32 mg/L), low activity (MIC = 64 mg/L) and no activity (MIC ≥ 128 mg/L). Compounds **2** and **3** displayed the highest activity against the Gram-negative bacterium *P. aeruginosa*. Higher MICs were obtained for the remaining purified compounds extracted from *A. dilatata* in tests with the pathogens.

Bromoageliferin (**2**) proved to be the most active against *P. aeruginosa* of the alkaloids isolated from *A. dilatata*. The MIC values for **2** and *P. aeruginosa* strains indicated high activity against ATCC 27853 (8 mg/L) and moderate activity against PAO1 (32 mg/L). To further study the activity of **2** against *P. aeruginosa*, 4 clinical isolates were included in the study. Bromoageliferin (**2**) displayed moderate activity against *P. aeruginosa* clinical isolates 29-200 SV, 30-127 VI, 30-223 SV and 30-230 SV, with a MIC value of 32 mg/L, while the corresponding MIC value for imipenem was 2 mg/L. Low concentrations of bromoageliferin (**2**) did not inhibit the growth of the tested strains of *A. baumannii* or *K. pneumoniae*. Additionally, **2** has also been reported to display antibacterial against Gram-positive bacteria, including *Micrococcus luteus* and *Bacillus subtilis* [22,28] and the human pathogen methicillin-resistant *Staphylococcus aureus* (MRSA) [29], and also against the Gram-negative bacteria *Escherichia coli* [22,28] and *Rhodospirillum salexigens* (a marine bacterium known to form biofilms) [30]. This work represents the first assessment of the antibacterial activity of bromoageliferin (**2**) against the multidrug resistant pathogen *P. aeruginosa*.

Also noteworthy is the activity of dibromoageliferin (**3**) against ATCC 27853 and PAO1 reference strains of *P. aeruginosa* (MIC, 32 mg/L) and against the two strains of *K. pneumoniae* included and the *A. baumannii* strain RYC 52763/97 (MIC, 64 mg/L). Ageliferin (**1**) showed a similar ability to inhibit growth against *K. pneumoniae* and *P. aeruginosa* (MIC, 64 mg/L). *A. baumannii* displayed greater resistance to these ageliferin-derived compounds.

Although **1** and **3** have previously been reported to display antibacterial activity against *M. luteus* [28], *B. subtilis* [22,24,28], *S. aureus* [24] and *E. coli* [22,24,28], this is the first report of an assessment of the antibacterial activity of **1** and **3** against the human pathogenic bacteria *A. baumannii*, *K. pneumoniae* and *P. aeruginosa*.

**Figure 2:**
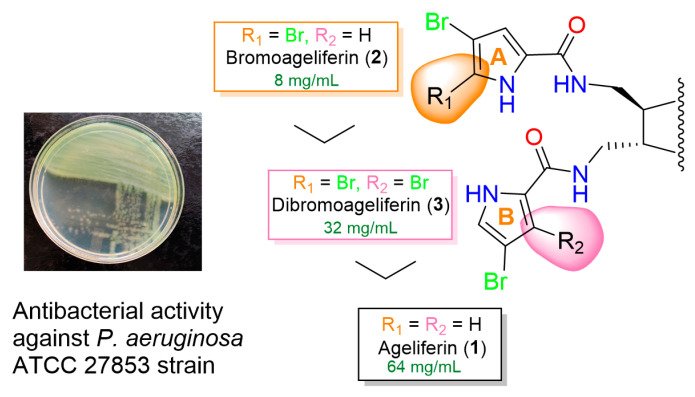
Influence of the presence of bromine atoms in A and B pyrrol rings in the antibacterial activity of compounds **1**–**3** against *P. aeruginosa* ATCC 27853 strain.

Isolation of the three ageliferins (**1**–**3**), which show slight structural differences, and assessment of their antibacterial activity allowed us to identify a relationship between the structure of the compounds and their activity against the *P. aeruginosa* ATCC 27853 strain. The structural comparison of the three ageliferins (**1**–**3**) in relation to the MIC values for this strain indicated that the presence of a second bromine atom at C-2 of pyrrol A ring increases the antibacterial activity. Thus, **1**, which does not contain the atom, was less active than **2** and **3**, in which the atom does occur (**1** was **eight** times less active than **2** and two times less active than **3**). However, the presence of a second bromine atom at C-2 of pyrrol B ring decreased the antibacterial activity because **2**, which bears a hydrogen at C-2 of the pyrrol B ring was more active than **3,** which bears a bromine atom at that position (**2** was four times more active than **3**) (Figure 2).

By contrast, the antibacterial analysis revealed that compounds **4**–**8** were moderately active or not active against all *A. baumannii*, *K. pneumoniae* and *P. aeruginosa* strains. However, these compounds have previously been shown to possess some antibacterial activity. Thus, **4** was reported to display antibacterial activity against *A. baumannii* [29], *P. aeruginosa* [23,27], *M. luteus* [28], *S. aureus* [23,24,27,29], *B. subtilis* [22,23,24,27,28], *B. cereus* [27], *Streptococcus faecalis* [27], *Salmonella typhi* [27] and *E. coli.* [22,24,27,28], but not against *S. aureus* (MRSA), *Mycobacterium intracellulare* or *M. tuberculosis* [31]. Compound **5** has been reported to be active against *B. subtilis* but not against *S. aureus* or *E. coli* [24]. Compound **6** did not display antimicrobial activity against *S. aureus*, *E. coli* or *Proteus vulgaris* [32]. Compound **7** has been reported to display activity against *S. aureus, E. coli, Serratia marcescens* and *Micrococcus* sp. [33], but not against *B. megaterium* [34], *B. subtilis* [35], *S. aureus* [35], *E. coli* [34,35] or *Mycobacterium smegmatis* [36]. Moreover, **7** did not display enzyme inhibitory activity in analogous FabI enzymes from *M. tuberculosis* (MtFabI, InhA) and *E. coli* (EcFabI) [37]. Compound **8** was reported inactive as it did not display antifouling activity and was unable to inhibit biofilm formation in the marine bacterial species *Pseudoalteromonas* spp. and *Paracoccus* sp. [38]. However, this is the first report of the evaluation of the antibacterial activity of **5**–**8** against *A. baumannii*, *K. pneumoniae* and *P. aeruginosa* and of **4** against *K. pneumoniae*. 

Taking into account the remarkable MIC values observed for bromoageliferin (**2**) against *P. aeruginosa*, which is particularly problematic in serious infections such as cystic fibrosis, we wished to gain further insight into the antibacterial activity of this compound by performing biofilm biomass inhibition analysis and a survival assay with *Galleria mellonella*.

### 2.3. Analysis of Biofilm Biomass Inhibition 

Bromoageliferin (**2**) has been reported to possess anti-biofilm activity against the marine R-proteobacterium *R. salexigens* [30]. Identification of a 2-aminoimidazole (2-AI) subunit in these bioactive brominated pyrrol alkaloids led to the suggestion that this structural motif, in tandem with the bicyclic core of bromoageliferin, may be the key pharmacophore that imparts biological activity [39]. For this reason, bromoageliferin (**2**) has been used as template for designing a library of simplified bromoageliferin scaffolds, such as *trans*-bromoageliferin analogue (TAGE, see Figure 1), which has proven to be very effective in inhibiting biofilm formation in strains of *P. aeruginosa* [39], *A. baumannii*, *Bordetella bronchiseptica* and *S. aureus* [40]. Furthermore, some of these simplified analogues suppress resistance of multiple antibiotic classes across a broad-spectrum of clinically important bacteria [40,41,42]. The parent natural product, bromoageliferin (**2**), was later reported to inhibit biofilm formation in two representative human pathogens, *A. baumannii* and *S. aureus* [29]. In the present study, we wished to evaluate, for the first time, the anti-biofilm activity of bromoageliferin (**2**) in *P. aeruginosa* strains.

**Figure 3:**
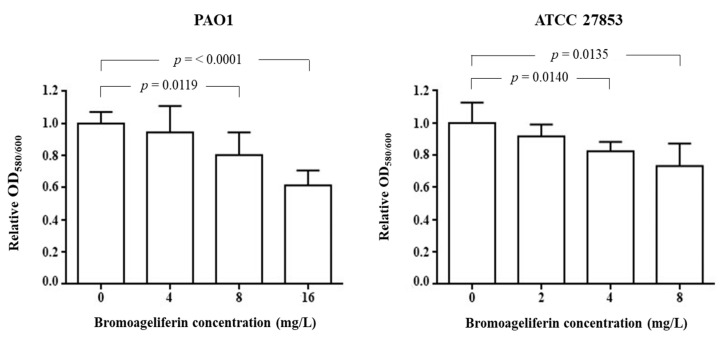
Quantification of biofilm formation after 24 h by *P. aeruginosa* strains PAO1 and ATCC 27853 in the presence of different concentrations of bromoageliferin (**2**).

The concentration-dependence of the effect of **2** on biofilm reduction formation was detected with both the PAO1 and ATCC 27853 reference strains (Figure 3). A significant decrease in the ability of *P. aeruginosa* PAO1 strain to generate biofilm was observed in the presence of bromoageliferin (**2**) at concentrations of 8 mg/L (*p* = 0.0119) and 16 mg/L (*p* ≤ 0.0001), relative to the control without compound. Regarding strain ATCC 27853, significant differences were found after addition of respectively 4 mg/L of **2** (*p* = 0.0140) or 8 mg/L (*p* = 0.0135) to the culture, relative to the control.

The data obtained regarding the inhibition of biofilm production by bromoageliferin (**2**) are consistent with those previously obtained with two simplified synthetic analogues of **2** against *P. aeruginosa* [39]. Indeed, a concentration of 100–200 µM of these analogues was required to inhibit 50% of biofilm production, while for **2** we observed 30–40% biofilm inhibition at concentrations of 8 or 16 mg/L (11.45 or 22.9 µM), depending on the *P. aeruginosa* strain used. Furthermore, inhibition of bacterial growth in the presence of the simplified synthetic analogues of bromoageliferin was evaluated by means of growth curves and was found to occur at 400–500 µM. Thus, the original compound **2** appears to have a greater capacity to inhibit bacterial growth, with MICs of 8–32 mg/L (11.45–45.83 µM) obtained in the present study.

Little is known about the antimicrobial mechanism of action of bromoageliferin (**2**). Although **2** displays high activity against *P. aeruginosa*, the growth inhibition is not as significant with the other pathogens tested, i.e., *A. baumannii* and *K. pneumoniae*. However, the inhibitory effect on biofilm production in different bacteria seems to be demonstrated. The genes encoding structural subunits of fimbriae *fimA* and *mfa1* of the oral pathogen *Porphyromonas gingivalis* show altered expression when the bacteria is grown in the presence of small molecules of bromoageliferin-derivates [43]. These molecules prevented *P. gingivalis* from binding to *Streptococcus gordoni* to form a mixed species biofilm community. The possible targets (*fimA* and *mfa1*) are involved in attachment and biofilm formation, which may partly explain the anti-virulence effect of bromoageliferin (**2**) observed in this study.

### 2.4. In Vivo Efficacy of Bromoageliferin against P. aeruginosa 

**Figure 4:**
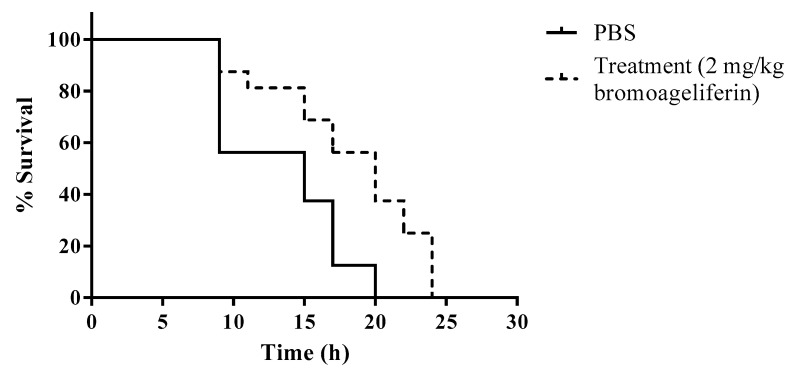
Survival of *G. mellonella* larvae (*n* = 15 per group) following infection with *P. aeruginosa* strain ATCC 27853 untreated (PBS) and treated with bromoageliferin (**2**) (2 mg/kg).

Mammalian animal models are considered the gold standard for screening new drugs. However, they have important ethical and administrative restrictions and are costly. The in vivo *Galleria* *mellonella* (wax moth) model is suitable for studying *P. aeruginosa* infections and the results obtained correlate well with those obtained in mammals. Although the wax moth does not have an adaptive immune system, it does possess an immune system analogous to the innate immune system in humans, and the model is therefore suitable for studying acute infections [44,45]. We therefore decided to test the in vivo efficacy of bromoageliferin (**2**) in a *G. mellonella* survival assay, in larvae infected with the *P. aeruginosa* ATCC 27853 strain (Figure 4). 

Although the survival rate to end point did not increase in larvae treated with bromoageliferin (**2**) relative to untreated larvae, a delay in death of the treated larvae was observed throughout the experiment. The mean survival time of larvae in the treated group was 18.3 h, compared with 13.5 h in the untreated larvae. Interestingly, at 20 h, once all untreated larvae were dead, a survival rate of 37.5% was observed in those treated with bromoageliferin (**2**). Therefore, significant differences were observed in mean survival time between treated and untreated larvae (*p* = 0.0035). Higher survival rates were not observed with higher concentrations of bromoageliferin (**2**) (5 and 20 mg/kg) (data not shown).

### 2.5. Additional Reported Activities for ***1***–***8***

In order to summarize the broad range of activities observed for **1**–**8**, we list here other previously reported biological activities.

Ageliferin (**1**) has previously been reported to act as an antiviral agent (*Herpes simplex* virus-type 1 and *Vesicular stomatitis*) [22], antifouling agent (*Balanus amphitrite amphitrite*) [22] and potent actomyosin ATPase activator [21], and also to display activity against the somatostatin receptor and vasoactive intestinal peptide (VIP) receptor [46]. By contrast, compound **1** did not display antifungal (*Penicillium atrovenetum* and *Saccharomyces* cerevisiae) [22], cytotoxic (*Artemia salina* [47] and monkey kidney cells [22] or antifouling activity (*Barnacle improvisus*) [47] and yielded a negative response in a biochemical prophage induction assay (PIA) [22].

Bromoageliferin (**2**) has previously been reported to act as an antiviral agent (*H. simplex*-type 1 and *V. stomatitis*) [22], potent actomyosin ATPase activator [21], inhibitor of voltage-operated, but not store-operated calcium entry in PC12 cells [48] and as a potent feeding deterrent (*Thalassoma bifasciatum*) [49]. Other biological studies of compound **2** report no antifungal activity (*P. atrovenetum* and *S. cerevisiae*) [22], cytotoxic activity (monkey kidney cells) [22] or activity in the biochemical prophage induction (BIA) assay [22] and also no antitumoral activity against three human tumor cell lines (A549 lung cancer cells, HT29 colonic cancer cells and MDA-MB-231 breast cancer cells) [50]. Compound **3** has been reported to display antiviral activity (*H. simplex* virus-type 1 and *V. stomatitis*) [22], potent actomyosin ATPase activity [21], to inhibit voltage-operated, but not store-operated calcium entry in PC12 cells [48], and to display potent feeding deterrent activity (*T. bifasciatum*) [49]. Dibromoageliferin (**3**) did not display antifungal activity (*P. atrovenetum* and *S. cerevisiae*) [22], cytotoxic activity (monkey kidney cells) [22], antitumoral activity against three human tumor cell lines (A549 lung cancer cells, HT29 colonic cancer cells and MDA-MB-231 breast cancer cells) [50] or activity in a biochemical prophage induction assay [22]. Sceptrin (**4**) displayed antiviral activity (*H. simplex* virus-type 1 and *V. stomatitis*) [22], activity in a biochemical prophage induction assay [22], antifouling activity (*B. amphitrite amphitrite*) [22], inhibitory activity against protein phosphatase type 2A [28], potent feeding deterrent activity (*T. bifasciatum*) [49], reduced voltage dependent calcium elevation in PC12 cells [48], antifungal activity (*Cryptococcus neoformans*) [31], fungicidal activity (*Phytophthora infestans* [51], *Candida albicans* [23], *Alternaria* sp. [23] and *Cladosporium cucumerinum* [23], and also inhibited cell motility in a variety of cancer cell lines (HeLa cells, metastatic breast cancer cell line (MDA-MB-231), lung cancer cell line (A549) and mouse fibroblasts (3T3) [52]. Sceptrin (**4**) also displayed antiparasitic activity (*Trypanosoma brucei rhodesiense* and *Plasmodium falciparum*) [53], activity at the somatostatin receptor and vasoactive intestinal peptide (VIP) receptor [46], antihistaminic activity (guinea pig ileum) [54], anti-muscarinic activity (muscarinic acetylcholine receptors (mAChR)) [55] and in the interaction with bacterial MreB protein [56]. However, sceptrin (**4**) did not display antifungal activity (*C. albicans*, *Aspergillus fumigatus* [31], *Stagonospora nodorum, Fusarium culmorum, Pyricularia grisei* and *Puccinia recondita* [51]), antimalarial activity (*P. falciparum*, D6 and W2 clone) [31], antiparasitic activity (*Leishmania donovani*) [31], antiviral activity (HIV-1 in PBM cells) [31], antifungal activity (*P. atrovenetum* and *S. cerevisiae*) [22], insecticidal activity (*Diabrotica virgifera virgifera, Heliothis virescens* and *Lygus hesperus*) [51], herbicidal activity (*Agrostis stolonifera* and *Nicotiana tabacum*) [51], cytotoxic activity (*A. salina*) [22], activity against monkey kidney cells [22], KB cell line [57], L6 cells [53], L929, KB-31, MCF-7, and FS4-LTM) [58], inhibitory activity in *P. falciparum* enzymes (PfFabI, PfFabG and PfFabZ) [53], antiparasitic activity (*Trypanosoma cruzi* and *L. donovani* [31,53] or antifouling activity (*B. improvisus*) [47]. Nakamuric acid (**5**) reduced the inhibition of cell motility in a variety of HeLa cancer cells lines [52]. 4-Bromo-1H-pyrrole-2-carboxylic acid (**6**) showed feeding deterrent activity (*T. bifasciatum*) [49] but no activity in reducing voltage dependent calcium elevation in PC12 cells [48], and also no cytotoxic (HL-60, K562, A549, and HCT-116 tumor cell lines) [32], no antitumoral activity against three human tumor cell lines (A549 lung cancer cells, HT29 colonic cancer cells and MDA-MB-231 breast cancer cells) [50] and no antimicrobial activity against *C. albicans* [32]. 4,5-Dibromopyrrole-2-carboxylic acid (**7**) displayed feeding deterrent activity (*T. bifasciatum*) [25,49,59], reduced voltage dependent calcium elevation in PC12 cells [48,60], enzyme inhibitory activity (PfFabI) [37], antiprotozoal activity (*P. falciparum*, *T. brucei rhodesiense*, *T. cruzi* and *L. donovani*) [37], immunosuppressive activity [61] and antifouling activity (*B. amphitrite*) [62]. However, compound **7** was not cytotoxic against rat skeletal myoblasts (L6 cells) [37], mouse lymphoma (L5178Y) [35], rat brain cancer (PC12) [35] or human cervix cancer cells (HeLa) [35] and did not display antitumoral activity against three human tumour cell lines (A549 lung cancer cells, HT29 colonic cancer cells and MDA-MB-231 breast cancer cells) [50], fungicidal activity (*Ustilago violacea*, *Mycotypha microspora*, *Eurotium repens*, *Fusarium oxysporum* [34], *S. cerevisiea*, *C. cucumerinum*, and *C. herbarum* [35], algicidal activity (*Chlorella fusca*) [34], activity in the protein kinase inhibition assays (cyclin-dependent kinase-1, cyclin-dependent kinase-5 and glycogen synthase kinase-3) [35], or inhibitory activity against 2,2-diphenyl-1-picrylhydrazyl (DPPH) radical scavenging, acetylcholinesterase (AChE) [63] and protein tyrosine phosphatase 1B (PTP1B) [36]. 

## 3. Materials and Methods 

### 3.1. General Experimental Chemical Procedures

Optical rotations were measured in a JASCO DIP-1000 polarimeter (JASCO, Tokyo, Japan), with a Na (589 nm) lamp and filter. ^1^H, ^13^C and 2D NMR spectra were recorded in a Bruker Avance 500 spectrometer, at 500 and 125 MHz, respectively, with CD_3_OD and D_2_O as solvents. HRESIMS experiments were performed in an Applied Biosystems QSTAR Elite system or a Thermo MAT95XP spectrometer. HPLC separations were performed in the Agilent 1100 liquid chromatography system equipped with a solvent degasser, quaternary pump, and diode array detector (Agilent Technologies, Waldbronn, Germany) with a semipreparative reversed phase column (Luna C18: 5 μ, 100 Å, 250 × 10 mm, Phenomenex, Lane Cove, Australia). Precoated silica gel plates (Merck, Kieselgel 60 F254, 0.25 mm, Merck Millipore, Merck KGaA, Darmstadt, Germany) were used for TLC analysis and the spots were visualized under a UV light (254 nm) or by heating the plate pretreated with H_2_SO_4_/H_2_O/AcOH (1:4:20).

### 3.2. Sponge Collection

The sponge *A. dilatata* was collected by SCUBA from the waters surrounding Cozumel Island, Quintana Roo (20°43′55.03″ N/87°00′24.70″ W), at depths ranging from 10 to 15 m, in October 2016. The sponges were frozen immediately after collection. A voucher specimen E25-1 was deposited in the Phylum Porifera Gerardo Green National Collection of the Institute of Marine Sciences and Limnology (ICMyL) at the National Autonomous University of Mexico (UNAM), Mexico City.

### 3.3. Extraction and Isolation 

Sliced bodies of *A. dilatata* (wet weight, 431.2 g; dry weight, 113.0 g) were exhaustively extracted with CH_3_OH-CH_2_Cl_2_ (1:1, 3 × 1.5 L) at room temperature. The combined extracts were concentrated under reduced pressure to yield 20.0 g of a crude residue that was first partitioned between CH_2_Cl_2_ and H_2_O (1:1 *v*/*v*). The resulting aqueous portion was extracted with *n*-butanol (200 mL) to yield the *n*-butanol fraction (3.25 g). The organic phase was concentrated under reduced pressure and partitioned between 10% aqueous CH_3_OH (400 mL) and hexane (2 × 400 mL) to produce 227.4 mg of the hexane fraction, after removal of the solvent under reduced pressure. The H_2_O content (% *v*/*v*) of the methanolic fraction was adjusted to 50% aqueous CH_3_OH, and this mixture was extracted with CH_2_Cl_2_ (100 mL) to yield 109.4 mg of the CH_2_Cl_2_ fraction and 150.4 mg of the remaining aqueous methanolic fraction, after removal of the solvent under reduced pressure. 

The *n*-butanol fraction was subjected to Solid Phase Extraction (SPE) with RP-18 (Merck KGaA) using a stepped gradient from H_2_O to CH_3_OH and then CH_2_Cl_2_. For separation, the fraction was eluted with H_2_O/CH_3_OH (2:1, 520 mg) by RP-HPLC with a mobile phase consisting of a gradient from 40% to 60% of CH_3_OH in H_2_O for 3 min (*v*/*v*, each containing 0.04% trifluoroacetic acid) followed by isocratic elution at 60% of CH_3_OH for 13 min and, finally, a gradient from 60% to 100% of CH_3_OH in H_2_O at a flow rate of 2.0 mL/min for 9 min, which yielded 3,7-dimethylisoguanine (**8**) (30.0 mg; *t*_R_ = 6.1 min), sceptrin (**4**) (5.8 mg; *t*_R_ = 10.2 min), ageliferin (**1**) (6.5 mg; *t*_R_ = 15.1 min), 4-bromo-1H-pyrrole-2-carboxylic acid (**6**) (10.0 mg; *t*_R_ = 16.6 min) and bromoageliferin (**2**) (2.3 mg; *t*_R_ = 23.8 min).

Separation of the fraction eluted with H_2_O/CH_3_OH (1:1, 250.6 mg) by RP-HPLC with a mobile phase consisting of a gradient from 40% to 50% of CH_3_OH in H_2_O (*v*/*v*, each containing 0.04% trifluoroacetic acid) for 2 min, followed by isocratic elution at 50% of CH_3_OH for 8 min, followed by a gradient from 50% to 60% of CH_3_OH in H_2_O for 9 min, isocratic elution at 60% of CH_3_OH for 8 min and, finally, a gradient from 60% to 100% of CH_3_OH in H_2_O at a flow rate of 2.0 mL/min for 13 min, which yielded 4-bromo-1H-pyrrole-2-carboxylic acid (**6**) (7.3 mg; *t*_R_ = 24.7 min), nakamuric acid (**5**) (3.0 mg; *t*_R_ = 26.5 min), bromoageliferin (**2**) (2.5 mg; *t*_R_ = 34.7 min) and 4,5-dibromopyrrole-2- carboxylic acid (**7**) (12.2 mg; *t*_R_ = 36.5 min).

Separation of the fraction eluted with H_2_O/CH_3_OH (1:2, 212.7 mg) by RP-HPLC with a mobile phase consisting of isocratic elution at 50% CH_3_OH in H_2_O (*v*/*v*, each containing 0.04% trifluoroacetic acid) for 5 min, followed by a gradient from 50% to 60% of CH_3_OH in H_2_O for 10 min, followed by isocratic elution at 60% of CH_3_OH for 10 min and, finally, a gradient from 60% to 100% of CH_3_OH in H_2_O at a flow rate of 2.0 mL/min for 15 min, which yielded 4-bromo-1H-pyrrole-2-carboxylic acid (**6**) (2.4 mg; *t*_R_ = 19.7 min), nakamuric acid (**5**) (1.8 mg; *t*_R_ = 20.8 min), bromoageliferin (**2**) (1.7 mg; *t*_R_ = 22.8 min) and 4,5-dibromopyrrole-2- carboxylic acid (**7**) (17.7 mg; *t*_R_ = 32.6 min).

The aqueous methanolic fraction (150.4 mg) was subjected to RP-HPLC separation with a mobile phase consisting of isocratic elution at 50% CH_3_OH in H_2_O (*v*/*v*, each containing 0.04% trifluoroacetic acid) for 5 min, followed by a gradient from 50% to 60% of CH_3_OH in H_2_O for 10 min, followed by a gradient from 60% to 65% of CH_3_OH in H_2_O and for 15 min, finally a gradient from 65% to 100% of CH_3_OH in H_2_O at a flow rate of 2.0 mL/min for 10 min, which yielded ageliferin (**1**) (2.6 mg; *t*_R_ = 17.3 min), 4-bromo-1H-pyrrole-2-carboxylic acid (**6**) (3.3 mg; *t*_R_ = 18.2 min), nakamuric acid (**5**) (3.6 mg; *t*_R_ = 19.3 min), bromoageliferin (**2**) (3.2 mg; *t*_R_ = 23.8 min), 4,5-dibromopyrrole-2- carboxylic acid (**7**) (4.2 mg; *t*_R_ = 29.2 min) and dibromoageliferin (**3**) (1.2 mg; *t*_R_ = 33.0 min).

### 3.4. Structural Characterization

**Ageliferin** (**1**). ${\left[\alpha \right]}_{D}^{25}$ +15.1° (c 0.2, CH_3_OH); ^1^H and ^13^C NMR see SM; (−)-HRESIMS *m/z* 617.0380 [M−H]^−^ (calcd. for C_22_H_23_^79^Br_2_N_10_O_2_, 617.0378).
**Bromoageliferin** (**2**). ${\left[\alpha \right]}_{D}^{25}$ +9.1° (c 0.2, CH_3_OH); ^1^H and ^13^C NMR see SM; (−)-HRESIMS *m/z* 694.9482 [M−H]^−^ (calcd. for C_22_H_22_^79^Br_3_N_10_O_2_, 694.9483).
**Dibromoageliferin** (**3**). ${\left[\alpha \right]}_{D}^{25}$ +4.0° (c 0.2, CH_3_OH); ^1^H and ^13^C NMR see SM; (−)-HRESIMS *m/z* 772.8582 [M−H]^−^ (calcd. for C_22_H_21_^79^Br_4_N_10_O_2_, 772.8588).
**Sceptrin** (**4**). ${\left[\alpha \right]}_{D}^{25}$ −13.8° (c 0.2, CH_3_OH); ^1^H and ^13^C NMR see SM; (+)-HRESIMS *m/z* 619.0531 [M−H]^+^ (calcd. for C_22_H_25_^79^Br_2_N_10_O_2_, 619.0523).
**Nakamuric acid** (**5**). ${\left[\alpha \right]}_{D}^{25}$ − 9.5° (c 0.2, CH_3_OH); ^1^H and ^13^C NMR see SM; (−)-HRESIMS *m/z* 579.9945 [M−H]^−^ (calcd. for C_20_H_20_^79^Br_2_N_7_O_4_, 579.9949).
**4-bromo-1H-pyrrole-2-carboxylic acid** (**6**). ^1^H and ^13^C NMR see SM; (−)-HRESIMS *m/z* 187.9353 [M−H]^−^ (calcd. for C_5_H_3_^79^BrNO_2_, 187.9353).
**4,5-dibromopyrrole-2- carboxylic acid** (**7**). ^1^H and ^13^C NMR see SM; (−)-HRESIMS *m/z* 265.8455 [M−H]^−^ (calcd. for C_5_H_2_^79^Br_2_NO_2_, 265.8458).
**3,7-dimethylisoguanine** (**8**). ^1^H and ^13^C see SM; (+)−HRESIMS *m/z* 180.0881 [M+H]^+^ (calcd. for C_7_H_10_N_5_O, 180.0880).

### 3.5. Bacterial Strains and Culture Media

Reference strains and clinical isolates of three Gram-negative pathogens *A. baumannii (*ATCC 17978 and RYC 52763/97 strains), *K. pneumoniae* (ATCC 700603 and KP 1803 strains) and *P. aeruginosa* (ATCC 27853, PAO1, 29-200 SV, 30-127 VI, 30-223 SV, and 30-230 SV strains) included in the study are listed in Table 2. Bacterial strains were frozen in Luria-Bertani (LB) with 10% glycerol and stored at −80 °C until analysis, when they were grown at 37 °C in LB medium. 

### 3.6. Microdilution Method: Minimum Inhibitory Concentration

The minimum inhibitory concentrations (MIC) of **1**–**8** were evaluated against bacterial strains by the microdilution method, as described by Clinical and Laboratory Standards Institute (CLSI), with some modifications [67]. Dimethylsulfoxide (DMSO) was used to dissolve the crude extracts, at a maximum concentration of 1.2% *v*/*v* in the well with the highest concentration of the plate (128 mg/L). Briefly, the strains were cultured overnight in Mueller Hinton II (MH) agar plates (Becton Dickinson) at 37 °C, and the turbidity of the bacterial suspensions was standardized at 0.5 on the McFarland scale to prepare the inocula. Wells were inoculated with approximately 1 × 10^6^ colony forming units/mL. Two-fold serial dilutions of compounds were performed in 96-wells microplates, in Mueller Hinton II broth medium (Sigma, St. Louis, MO, USA). The range of extract concentrations used for MIC analysis was 0.5–128 mg/L. One well was used in each row as positive growth control, composed of growth media and bacterial suspension, and another well, used as a negative control, consisted of medium without bacterial inoculum. Solvent controls of DMSO were included to determine whether the used concentration interfered with bacterial growth. The β-lactam antibiotic imipenem, which displays a broad spectrum of activity against Gram-negative bacteria, was used as a control for the microdilution methodology. The minimum inhibitory concentration was determined after incubation for 20–24 h at 37 °C and was established as the lowest concentration of the compound in which the bacterial strains did not grow. All extracts were tested in triplicate.

### 3.7. Biofilm Inhibition Assay

*P. aeruginosa* strains ATCC 27853 and PAO1 were cultivated on MH agar for 18 h at 37 °C and used to inoculate 5 mL of MH broth. These cultures were, in turn, grown overnight at 37 °C with shaking. A 1:100 dilution of each strain (initial inoculum of approx. 1 × 10^7^ CFU/mL) was then incubated for 24 h in 24-well plates. Assays were performed in the presence of sub-MICs of bromoageliferin.

Bacterial growth was then measured at OD_600nm_, in an Epoch 2 Microplate Spectrophotometer (BioTek Instruments, VT, USA), to determine the total cell biomass. Afterward, medium with bacteria was removed from the wells and then, they were washed with phosphate-buffered saline (PBS). Biofilm formation was determined by staining with a final concentration of 10% crystal violet per well, washing vigorously with PBS and solubilizing in 30% (*v*/*v*) acetic acid. The OD_580nm_/OD_600nm_ ratio was calculated to normalize the amount of produced biofilm to the total cell biomass, thus avoiding variations due to different culture conditions. A minimum of 5 replicates were analyzed per condition. A Student’s *t*-test was carried out with GraphPad Prism (GraphPad Software, San Diego, CA, USA), in order to evaluate the statistical significance of observed differences (*p* ≤ 0.05).

### 3.8. Galleria Mellonella Treatment 

The efficacy of bromoageliferin (**2**) treatment was evaluated in a *G. mellonella* survival assay, as previously described [68]. The larvae were obtained from BioSystems Technology. Briefly, *P. aeruginosa* ATCC 27853 was grown to an OD_600nm_ of 0.7 at 37 °C, centrifuged, washed and resuspended in sterile PBS. Two groups of 15 larvae were injected with 10 µL of bacterial suspension containing 5 × 10^2^ CFU/mL. The treatment evaluated was bromoageliferin (2 mg/kg), and the same volume of sterile PBS was used as control. Higher bromoageliferin concentrations (5 and 20 mg/kg) were also tested. The groups were incubated at 37 °C in darkness, and survival was monitored during a period of 30 h. The resulting survival curves were plotted using the Kaplan-Meier method and analysed using the log-rank (Mantel-Cox) test.

## 4. Conclusions

We describe the isolation and structural characterization of eight known alkaloids **1**–**8**, most of which are bromopyrrols, isolated from specimens of the sponge *A. dilatata* collected on Cozumel Island (Yucatan Peninsula, Mexico), in the first chemical assessment of the secondary metabolites from this sponge. Although the antibacterial activity of these compounds has previously been reported, the activity of most of the compounds was evaluated for the first time in relation to problematic multidrug resistant pathogens, i.e., the Gram-negative bacteria *K. pneumoniae*, *P. aeruginosa* and *A. baumannii*. Bromoageliferin (**2**) and dibromoageliferin (**3**) display both moderate antibacterial activity against the *P. aeruginosa* PAO1 strain while they show high and moderate antibacterial activity, respectively, against the *P. aeruginosa* ATCC27853 strain. Compound **2** inhibited growth and biofilm production in *P. aeruginosa*, and increased the survival time of larvae in the in vivo *G. mellonella* assay. Moreover, a relationship between structure and activity was deduced from the antibacterial analysis of the three isolated ageliferins with similar structures (**1**–**3**). In summary, in vitro and in vivo findings for the multidrug resistant pathogen *P. aeruginosa* indicate bromoageliferin (**2**) as a promising lead for optimization in the design of new antimicrobial therapies.

## Figures and Tables

**Table 1:** Minimum inhibitory concentrations (mg/L) (MICs) of **1**–**8** and imipenem as control for reference strains of Gram-negative bacteria.

Compound	*Acinetobacter baumannii*		*Klebsiella pneumoniae*		*Pseudomonas aeruginosa*	
	ATCC 17978	RYC 52763/97	ATCC 700603	KP 1803	ATCC 27853	PAO1
Ageliferin (**1**)	≥128	≥128	64	64	64	64
Bromoageliferin (**2**)	≥128	≥128	≥128	≥128	8	32
Dibromoageliferin (**3**)	≥128	64	64	64	32	32
Sceptrin (**4**)	≥128	≥128	64	64	128	128
Nakamuric acid (**5**)	≥128	≥128	≥128	≥128	≥128	≥128
4-Bromo-1H-pyrrole-2-carboxylic acid (**6**)	≥128	≥128	≥128	≥128	64	≥128
4,5-Dibromopyrrole-2- carboxylic acid (**7**)	≥128	≥128	64	≥128	64	≥128
3,7-Dimethylisoguanine (**8**)	≥128	≥128	64	64	64	128
Imipenem (control)	0.5	16	0.25	2	2	2

**Table 2:** Reference strains and clinical isolates used in this work.

Bacterial Strain	Description	Source/References
*A. baumannii*		
ATCC 17978	Reference strain, completely sequenced.	ATCC ^a^
RYC 52763/97	Clinical isolate from respiratory tract.	Outbreak in Ramón y Cajal Hospital, Madrid, Spain [64]
*K. pneumoniae*		
ATCC 700603	Reference strain, completely sequenced.	ATCC
KP 1803	Clinical isolate from urinary tract, completely sequenced.	Outbreak in A Coruña Hospital, Spain [65]
*P. aeruginosa*		
ATCC 27853	Reference strain, completely sequenced.	ATCC
PAO1	Reference strain, completely sequenced.	ATCC
29-200 SV	Clinical isolate from respiratory tract.	MagicBullet clinical trial [66]
30-127 VI	Clinical isolate from respiratory tract.	MagicBullet clinical trial [66]
30-223 SV	Clinical isolate from respiratory tract.	MagicBullet clinical trial [66]
30-230 SV	Clinical isolate from respiratory tract.	MagicBullet clinical trial [66]

*^a^* American Type Culture Collection.

## Supplementary Materials

The following are available online at https://www.mdpi.com/1660-3397/18/6/326/s1, Figures S1–S24: NMR spectra in CD_3_OD and D_2_O, and HRESIMS of compounds **1**–**8**.
