# Supplementary material for: In Vitro and In Vivo Assessment of the Efficacy of Bromoageliferin, an Alkaloid Isolated from the Sponge Agelas dilatata, against Pseudomonas aeruginosa
Source: Mar Drugs. 2020 Jun 23;18(6):326. doi: 10.3390/md18060326 (PMC7345159; doi:10.3390/md18060326)
Supplement: Supplementary file 1 [file marinedrugs-18-00326-s001.pdf]

Supplementary Material

In Vitro and in Vivo Assessment of the Efficacy of Bromoageliferin, an Alkaloid Isolated from the Sponge *Agelas dilatata,* against *Pseudomonas aeruginosa*

Dawrin Pech-Puch[1], Mar Pérez-Povedano[1], Marta Martínez-Guitián[2], Cristina Lasarte-Monterrubio[2], Juan Carlos Vázquez-Ucha[2], German Bou[2], Jaime Rodríguez[1,*], Alejandro Beceiro[2,*,†] and Carlos Jiménez[1,*,†]

[1] Centro de Investigacións Científicas Avanzadas (CICA) e Departamento de Química, Facultade de Ciencias, AE CICA-INIBIC, Universidade da Coruña, 15071 A Coruña, Spain; dawrin.j.pech@udc.es (D.P.-P.); perezpovedanomaranabel@gmail.com (M.P.-P.)

[2] Servicio de Microbioloxía. Instituto de Investigación Biomédica, AE CICA-INIBIC Complexo Hospitalario Universitario A Coruña, 15006, A Coruña, Spain; m.martinez.guitian@gmail.com (M.M.-G.); crlasarm@gmail.com (C.L.-M.); juan.vazquez@udc.es (J.C.V.-U.); German.Bou.Arevalo@sergas.es (G.B.)

* Correspondence: jaime.rodriguez@udc.es (J.R.); alejandro.beceiro.casas@sergas.es (A.B.); carlos.jimenez@udc.es (C.J.)

† Same contribution to this work

INDEX

Figure S1-S2. NMR spectra data of compound **1** in CD_3OD……………….……………S3

Figure S3. **(-)-**HRESIMS of compound **1**……….……………………………………......S4

Figure S4-S5. NMR spectra data of compound **2** in CD_3OD……………………...….......S5

Figure S6. **(-)-**HRESIMS of compound **2**……….……………………………………......S6

Figure S7-S8. NMR spectra data of compound **3** in CD_3OD……………………...….......S7

Figure S9. **(-)-**HRESIMS of compound **3**……….……………………………………......S8

Figure S10-S11. NMR spectra data of compound **4** in CD_3OD………………..…..….......S9

Figure S12. **(+)-**HRESIMS of compound **4**……….…………………....……………......S10

Figure S13-S14. NMR spectra data of compound **5** in CD_3OD………………...…........S11

Figure S15. **(-)-**HRESIMS of compound **5**……….……………………………...….......S12

Figure S16-S17. NMR spectra data of compound **6** in CD_3OD………………..…..........S13

Figure S18. **(-)-**HRESIMS of compound **6**……….………………………………….......S14

Figure S19-S20. NMR spectra data of compound **7** in CD_3OD…………………….........S15

Figure S21. **(-)-**HRESIMS of compound **7**……….……………………..……........S16

Figure S22-S23. NMR spectra data of compound **8** in D_2O………..………..…...…......S17

Figure S24. **(+)-**HRESIMS of compound **8**……….………………………..…......S18

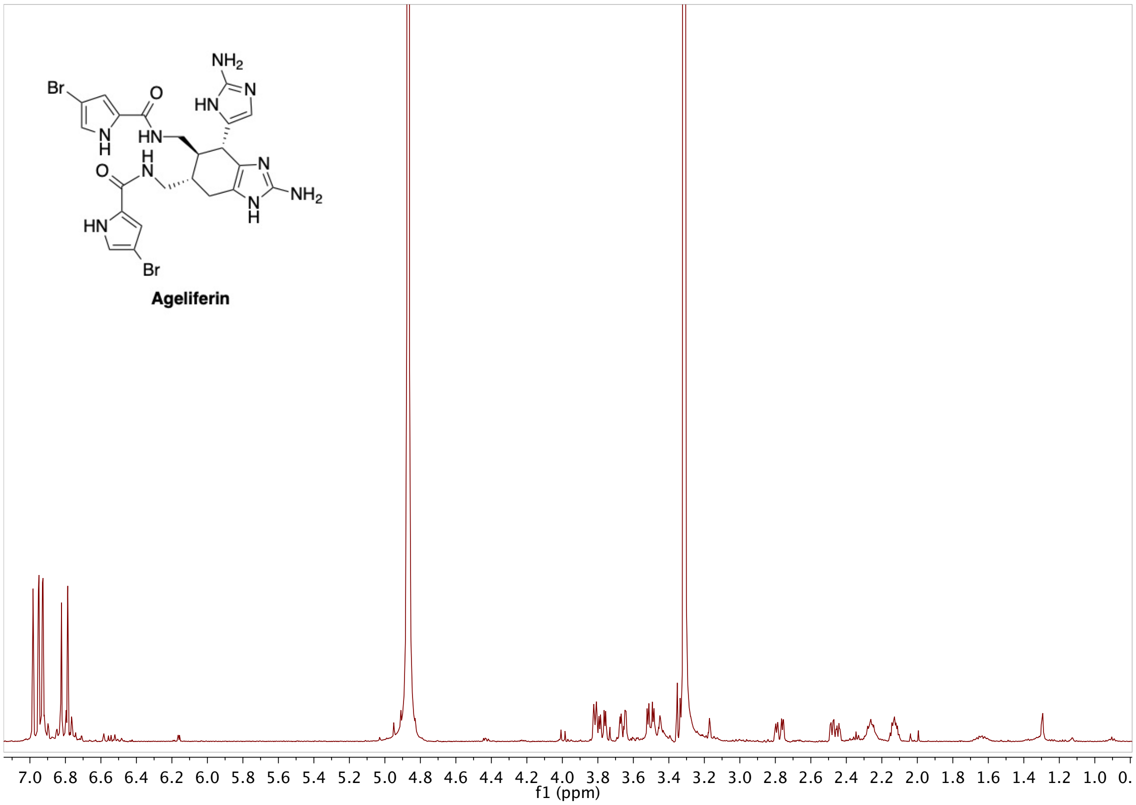

Figure S1. ^{1}H NMR spectrum of **1** (500 MHz, CD_3OD).

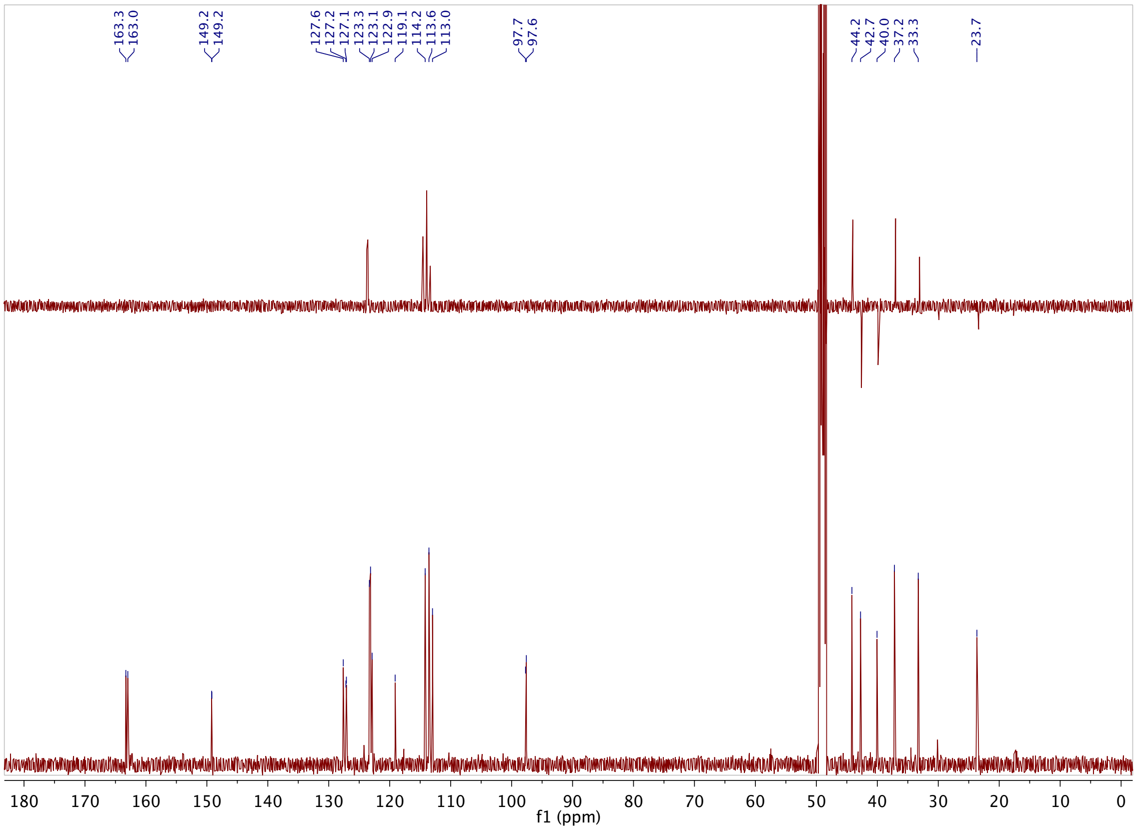

Figure S2. ^{13}C NMR and DEPT-135 spectra of **1** (125 MHz, CD_3OD).

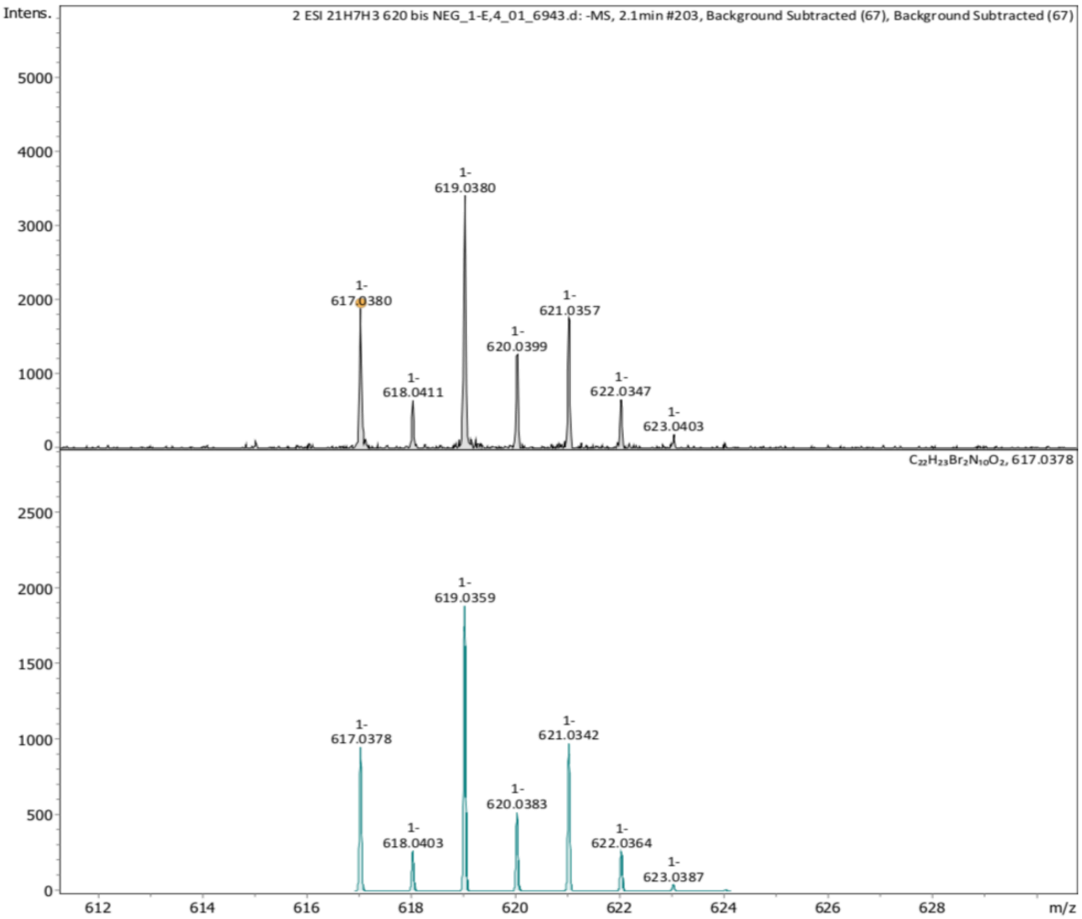

Meas. m/z	#	Ion Formula	Score	m/z	err [mDa]	err [ppm]	mSigma	rdb	e⁻ Conf	N-Rule
617.0380	1	C22H23Br2N10O2	100.00	617.0378	-0.2	-0.4	51.0	19.0	even	ok
	2	C23H29Br2N3O7	86.31	617.0378	-0.2	-0.4	55.8	13.5	odd	ok

Figure S3. (-)-HRESIMS of **1**.

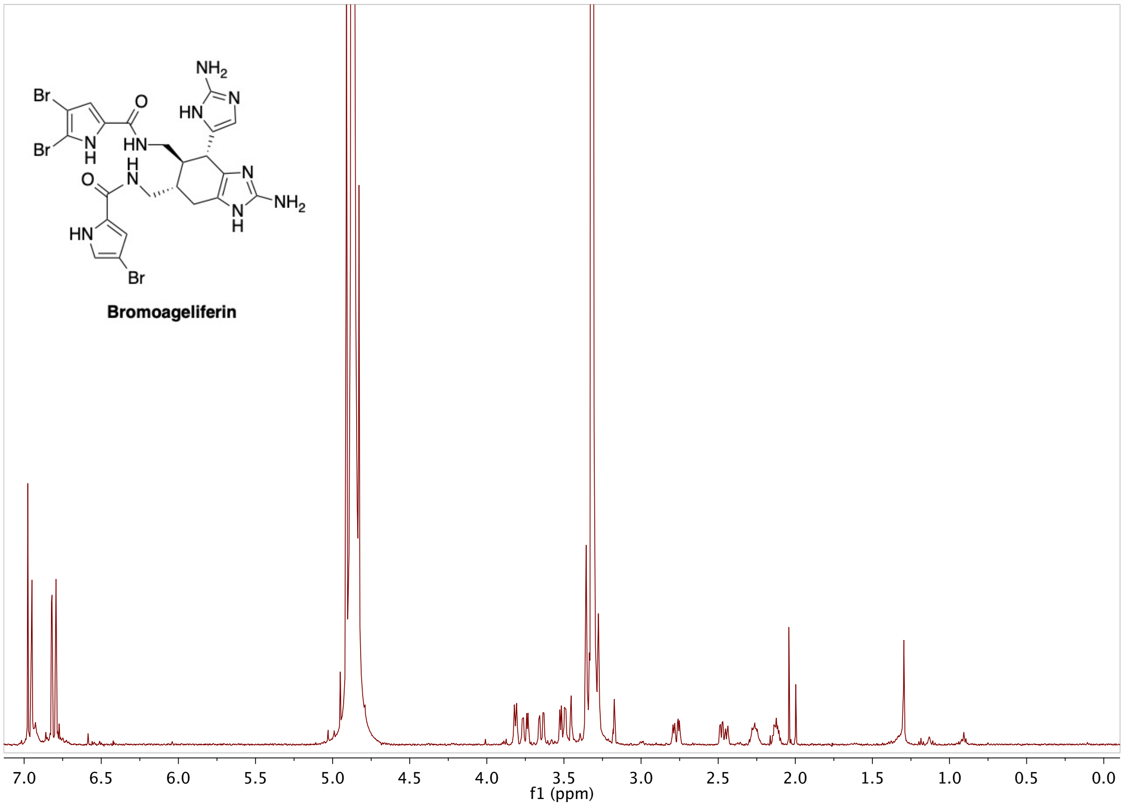

Figure S4. ^{1}H NMR spectrum of **2** (500 MHz, CD_3OD).

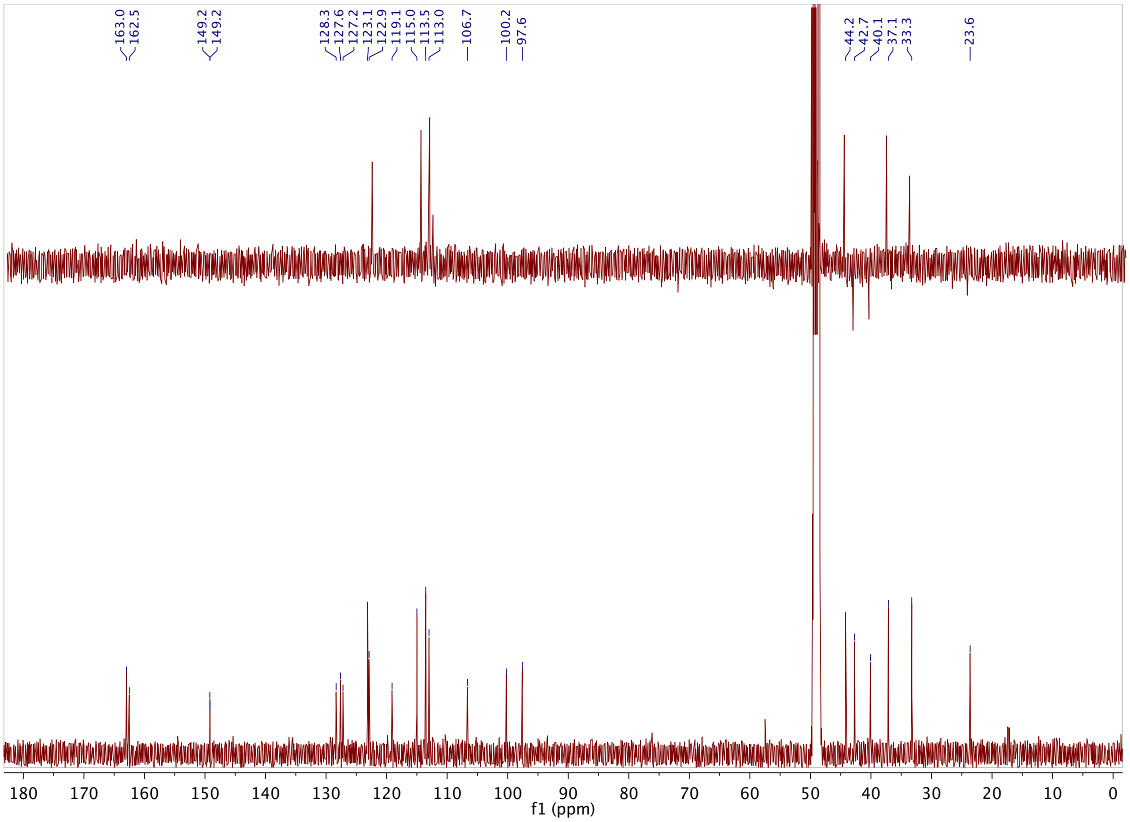

Figure S5. ^{13}C NMR and DEPT-135 spectra of **2** (125 MHz, CD_3OD).

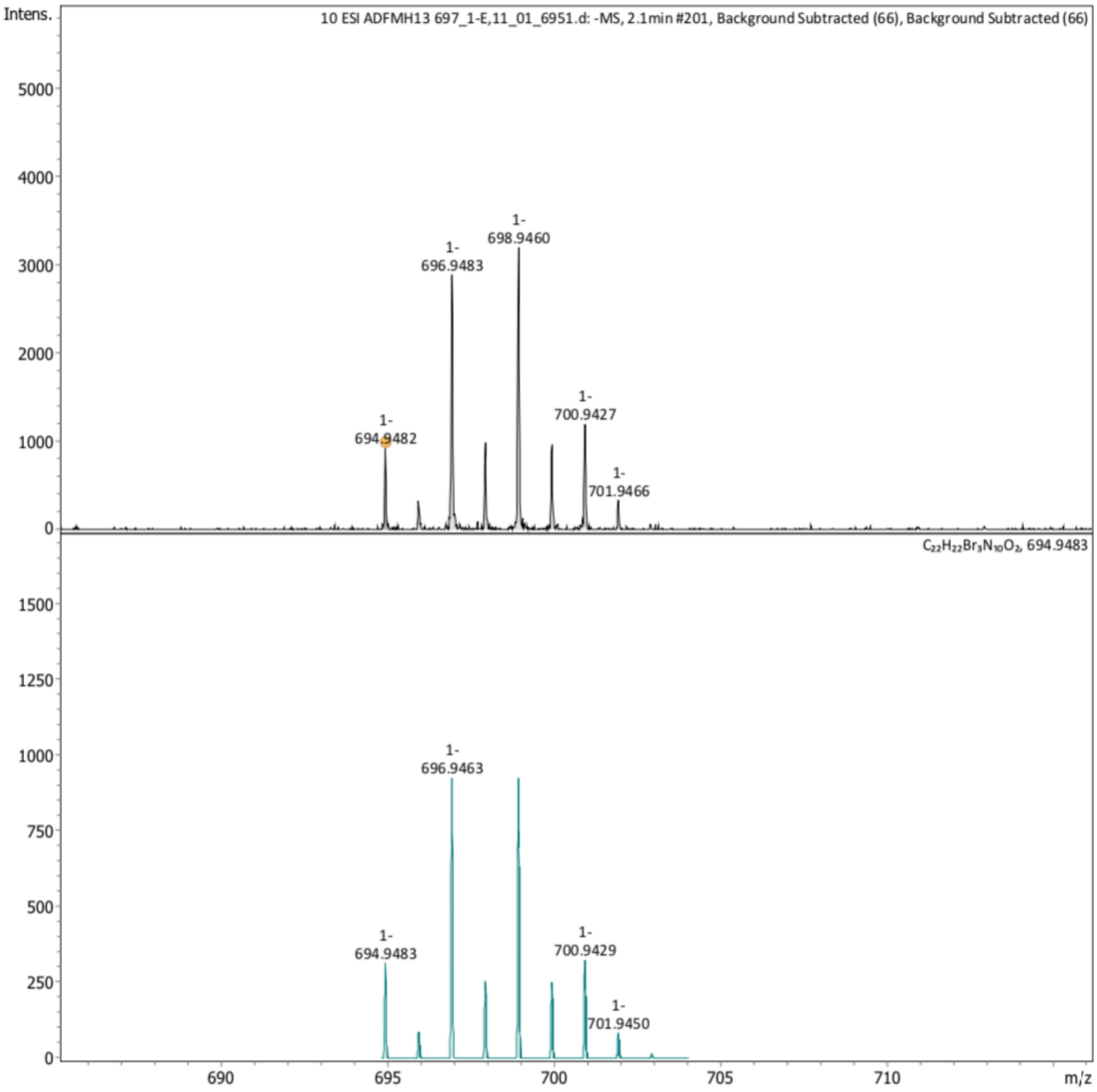

Meas. m/z	#	Ion Formula	Score	m/z	err [mDa]	err [ppm]	mSigma	rdb	e⁻ Conf	N-Rule
694.9482	1	C23H28Br3N3O7	100.00	694.9483	0.1	0.2	41.7	15.5	odd	ok
	2	C22H22Br3N10O2	86.11	694.9483	0.1	0.2	42.9	21.0	even	ok

Figure S6. (-)-HRESIMS of **2**.

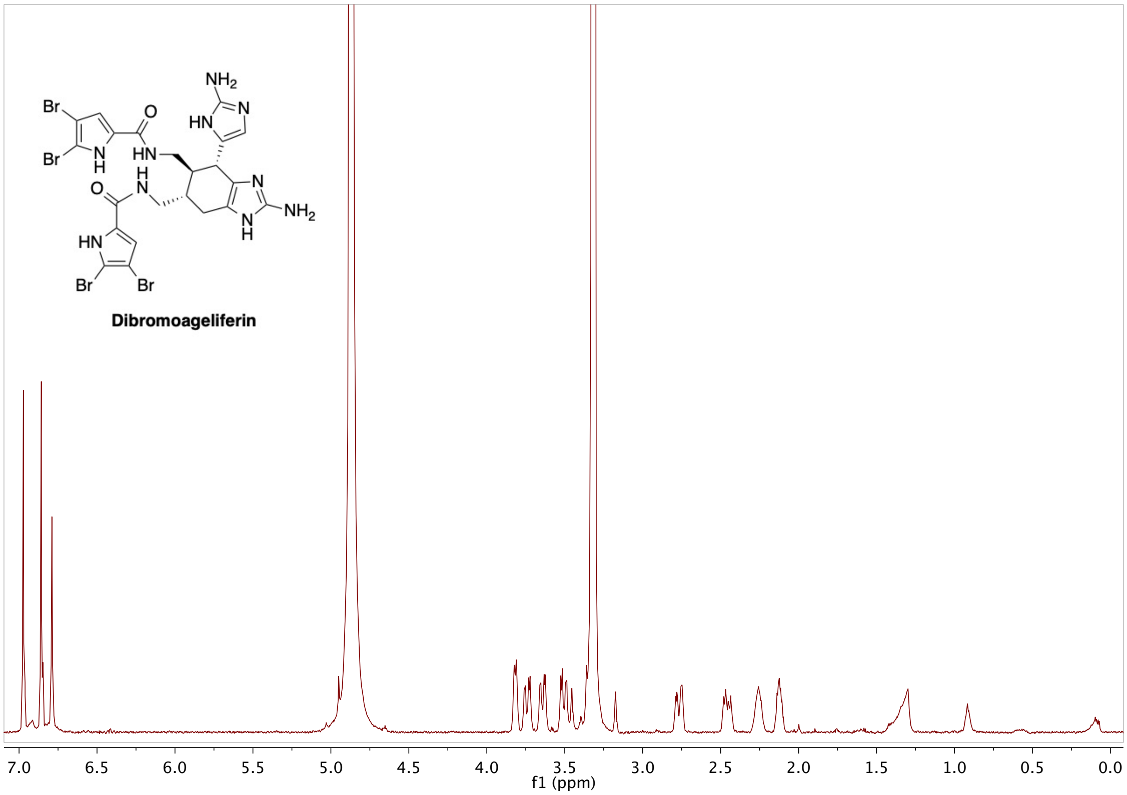

Figure S7. ^{1}H NMR spectrum of **3** (500 MHz, CD_3OD).

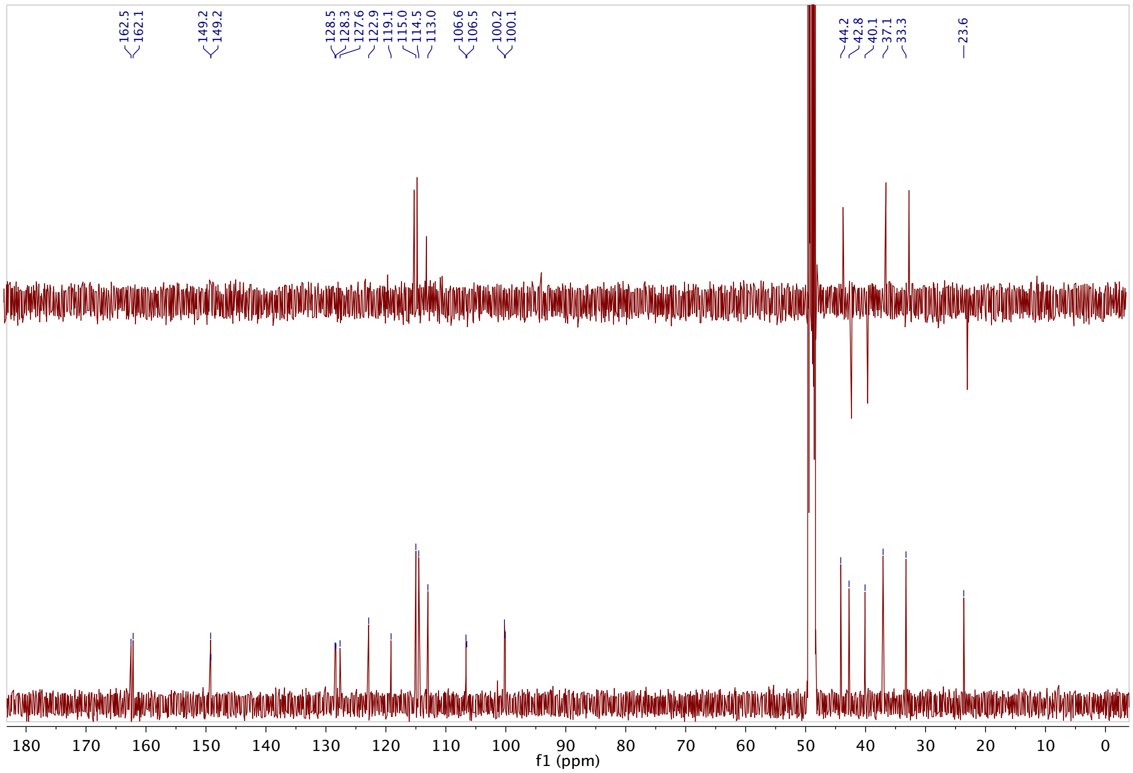

Figure S8. ^{13}C NMR and DEPT-135 spectra of **3** (125 MHz, CD_3OD).

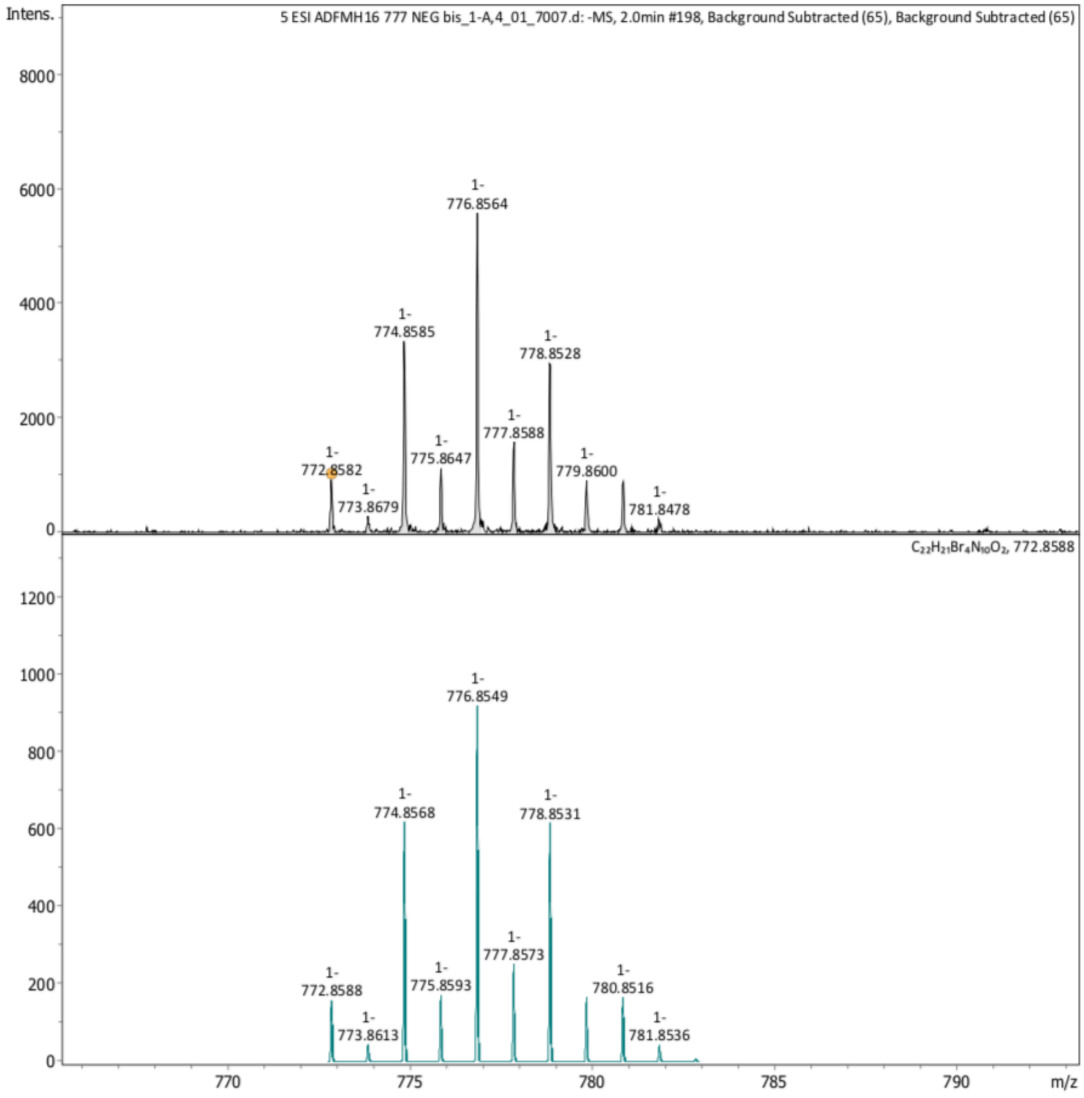

Meas. m/z	#	Ion Formula	Score	m/z	err [mDa]	err [ppm]	mSigma	rdb	e⁻ Conf	N-Rule
772.8582	1	C20H19Br4N13O	94.77	772.8575	-0.7	-1.0	52.5	23.5	odd	ok
	2	C18H17Br4N16	39.54	772.8561	-2.1	-2.7	52.6	24.0	even	ok
	3	C22H21Br4N10O2	100.00	772.8588	0.6	0.8	52.8	23.0	even	ok
	4	C24H23Br4N7O3	42.17	772.8601	2.0	2.5	53.6	22.5	odd	ok
	5	C23H27Br4N3O7	95.59	772.8588	0.6	0.8	54.2	17.5	odd	ok

Figure S9. (-)-HRESIMS of **3**.

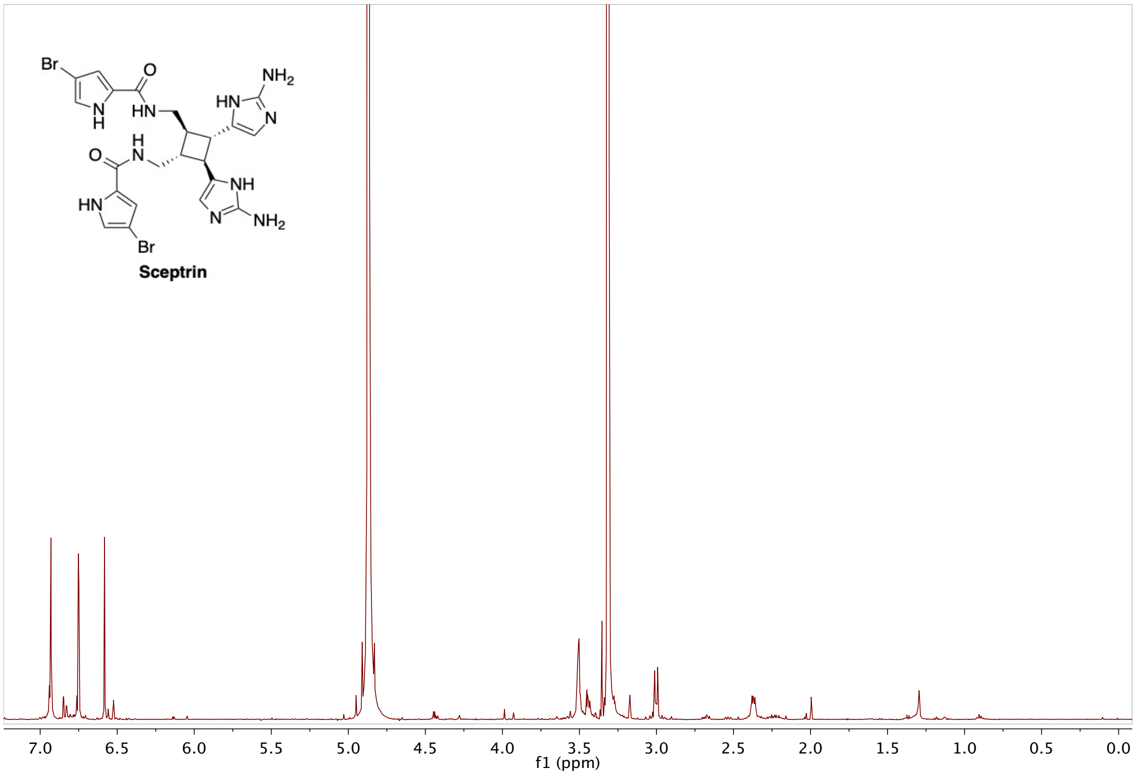

Figure S10. ^{1}H NMR spectrum of **4** (500 MHz, CD_3OD).

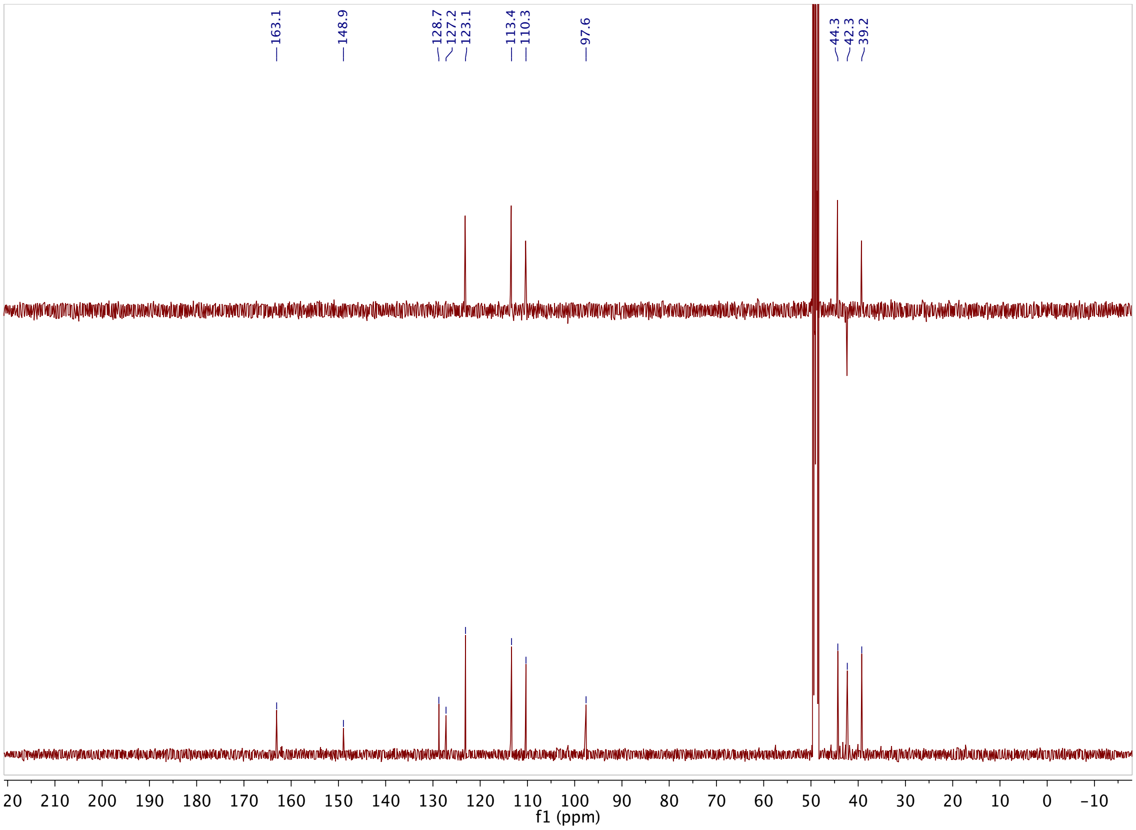

Figure S11. ^{13}C NMR and DEPT-135 spectra of **4** (125 MHz, CD_3OD).

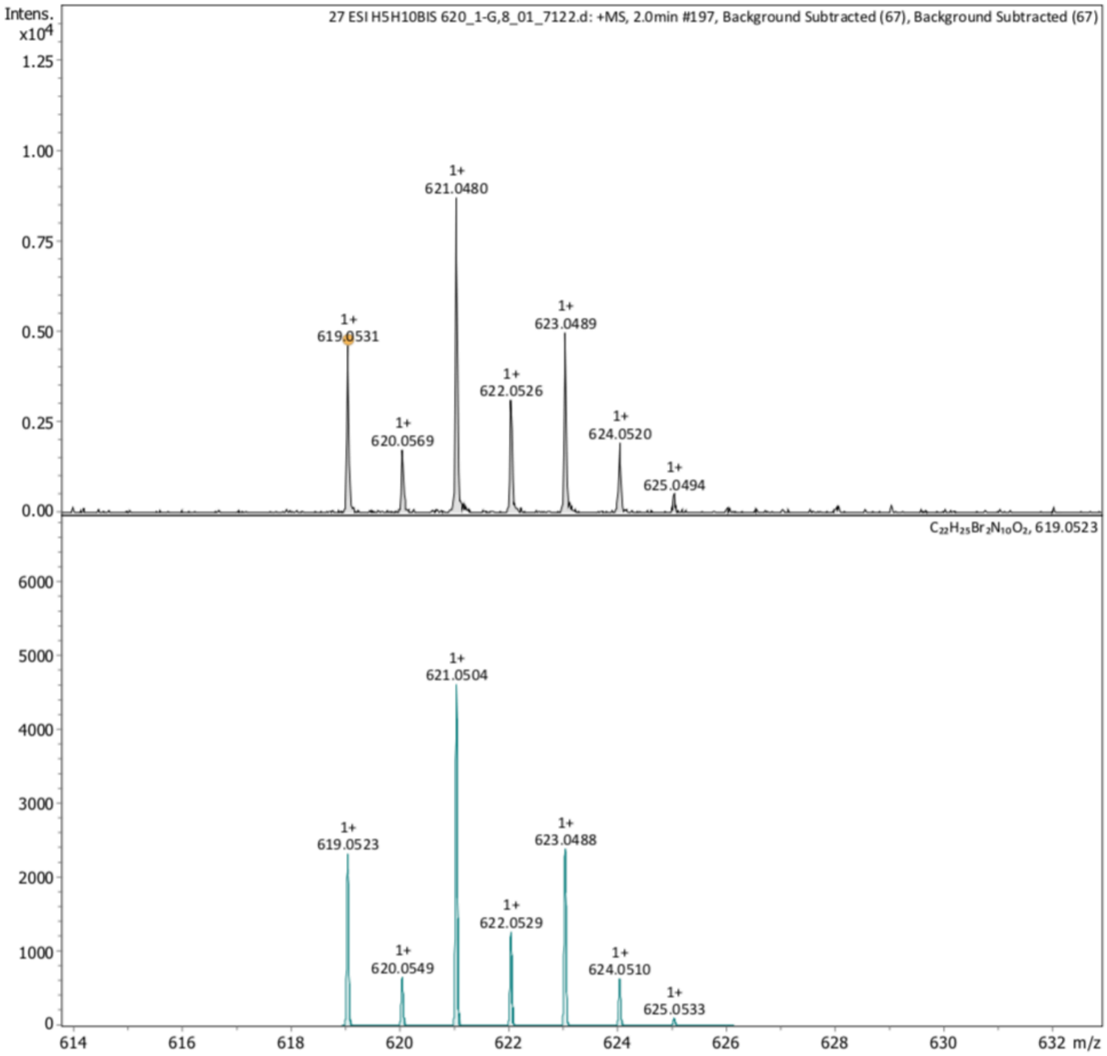

Meas. m/z	#	Ion Formula	Score	m/z	err [mDa]	err [ppm]	mSigma	rdb	e⁻ Conf	N-Rule
619.0531	1	C26H29Br2N4O4	50.48	619.0550	1.9	3.1	47.1	18.0	even	ok
	2	C24H27Br2N7O3	100.00	619.0537	0.6	0.9	52.0	18.5	odd	ok
	3	C25H33Br2O8	89.48	619.0537	0.6	0.9	55.4	13.0	even	ok
	4	C22H25Br2N10O2	77.93	619.0523	-0.8	-1.2	56.9	19.0	even	ok
	5	C23H31Br2N3O7	69.50	619.0523	-0.8	-1.2	60.4	13.5	odd	ok

Figure S12. (+)-HRESIMS of **4**.

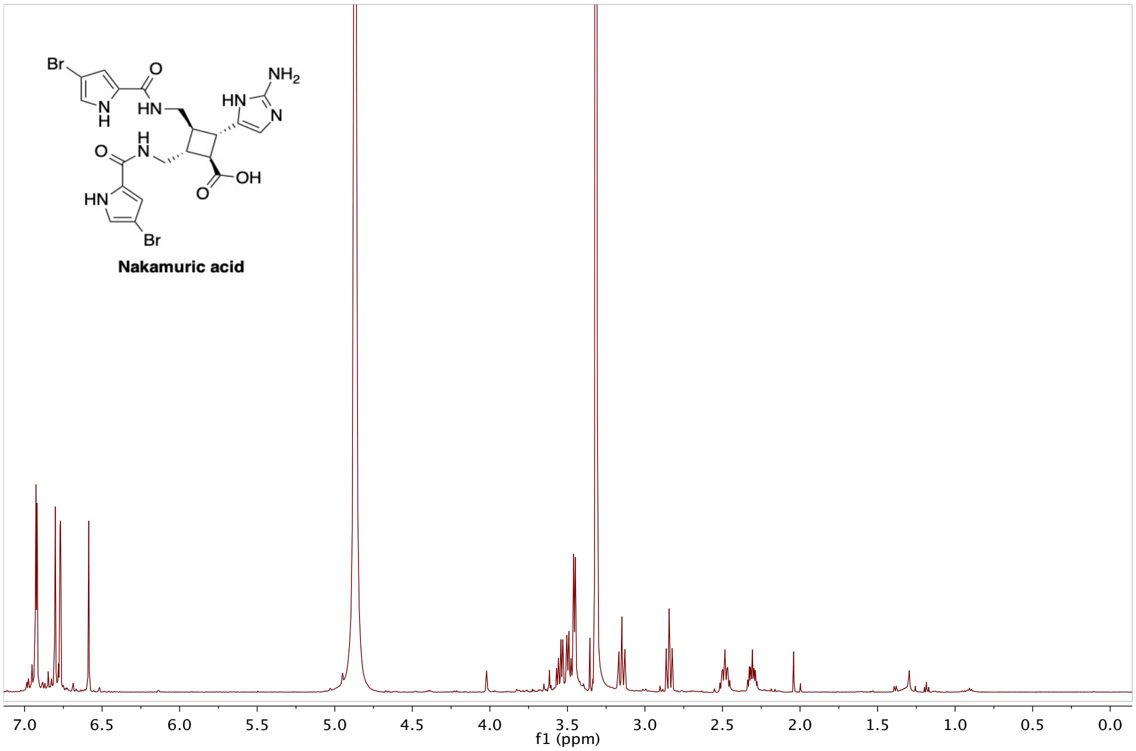

Figure S13. ^{1}H NMR spectrum of **5** (500 MHz, CD_3OD).

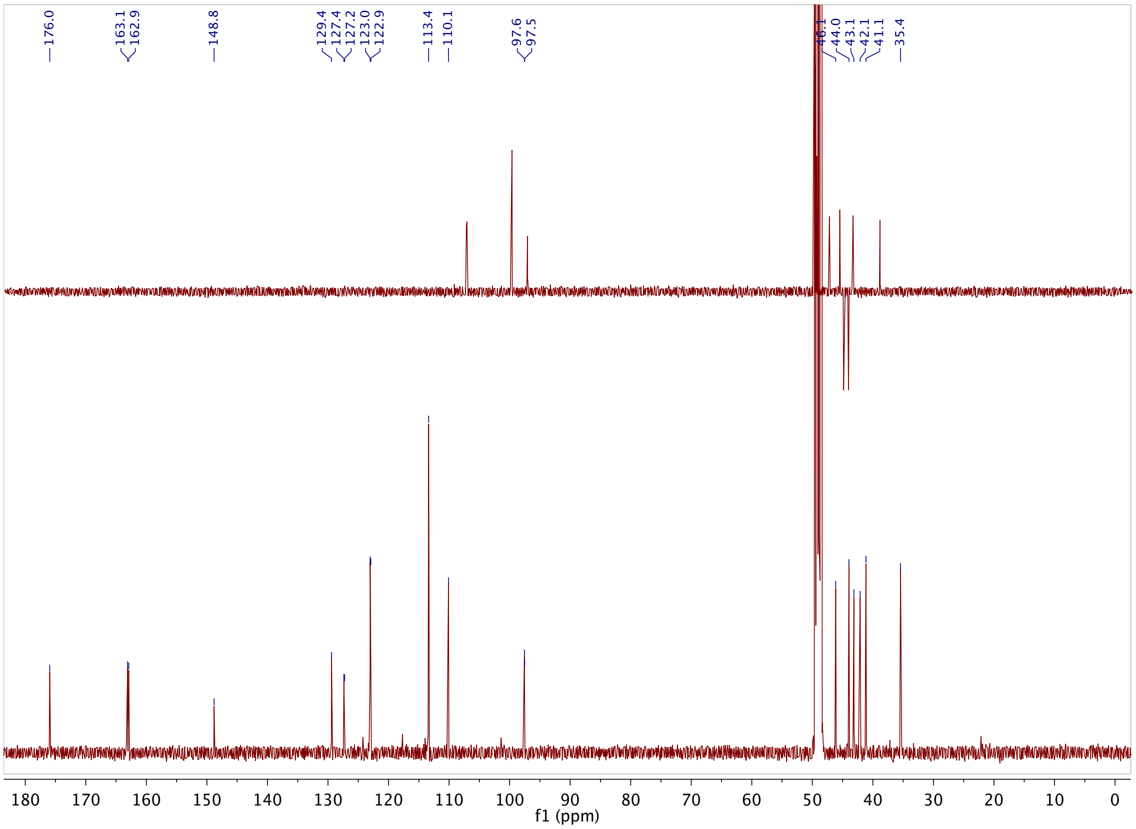

Figure S14. ^{13}C NMR and DEPT-135 spectra of **5** (125 MHz, CD_3OD).

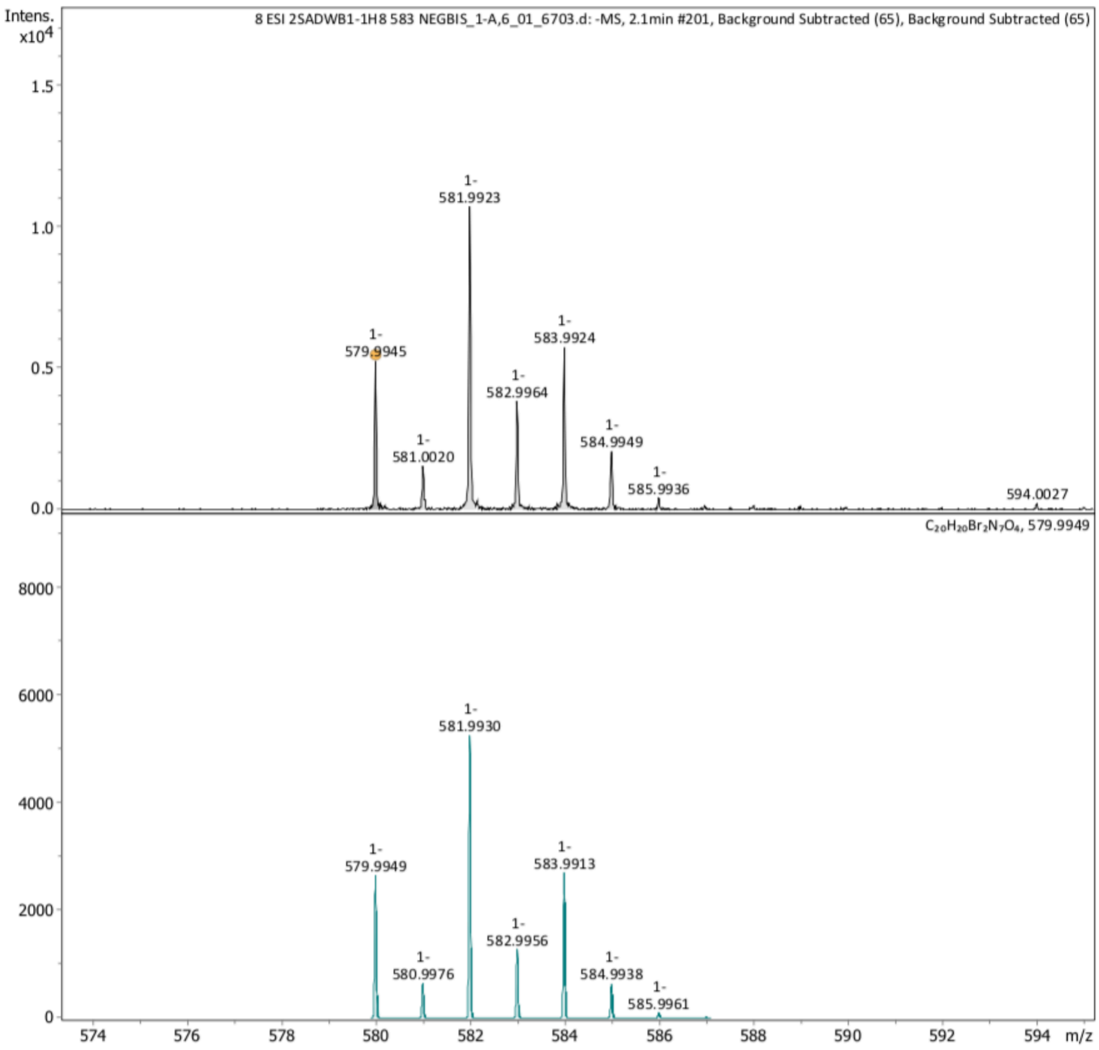

Meas. m/z	#	Ion Formula	Score	m/z	err [mDa]	err [ppm]	mSigma	rdb	e⁻ Conf	N-Rule
579.9945	1	C20H20Br2N7O4	100.00	579.9949	0.4	0.6	52.2	17.0	even	ok
	2	C21H26Br2O9	86.64	579.9949	0.4	0.6	56.6	11.5	odd	ok
	3	C18H18Br2N10O3	61.72	579.9936	-1.0	-1.7	57.4	17.5	odd	ok
	4	C19H24Br2N3O8	53.34	579.9936	-1.0	-1.7	61.8	12.0	even	ok

Figure S15. (-)-HRESIMS of **5**.

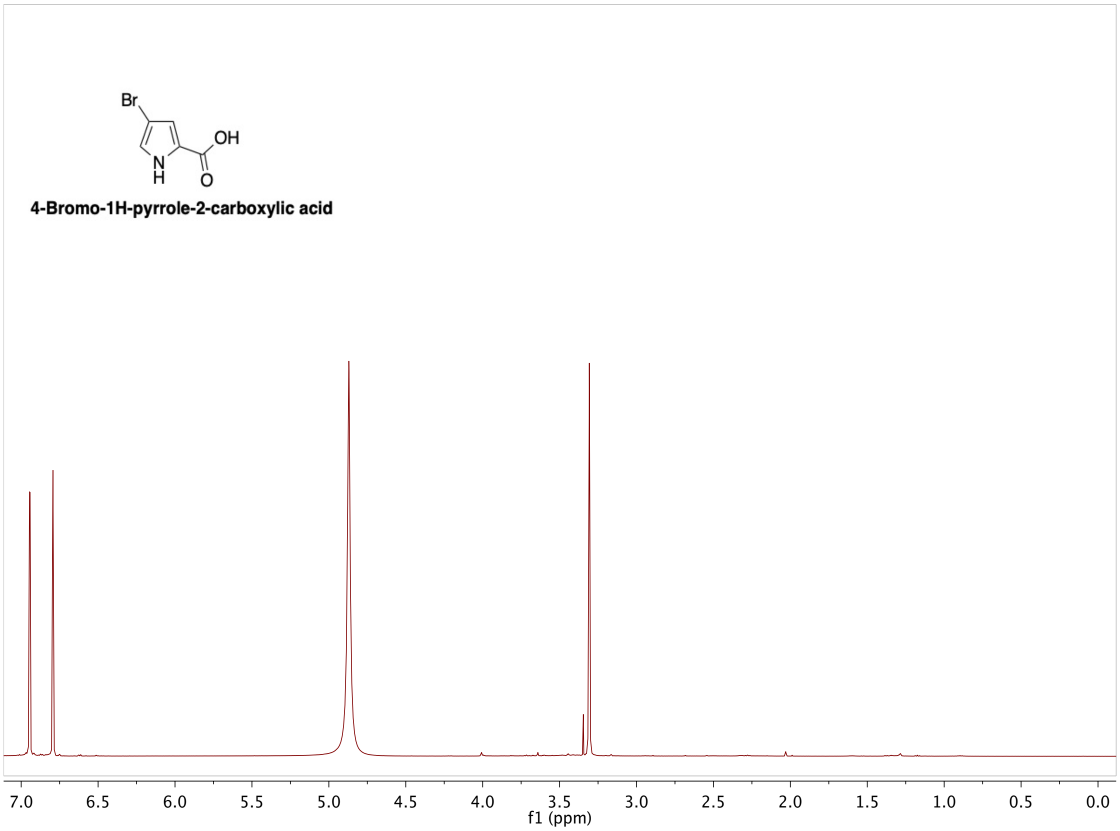

Figure S16. ^{1}H NMR spectrum of **6** (500 MHz, CD_3OD).

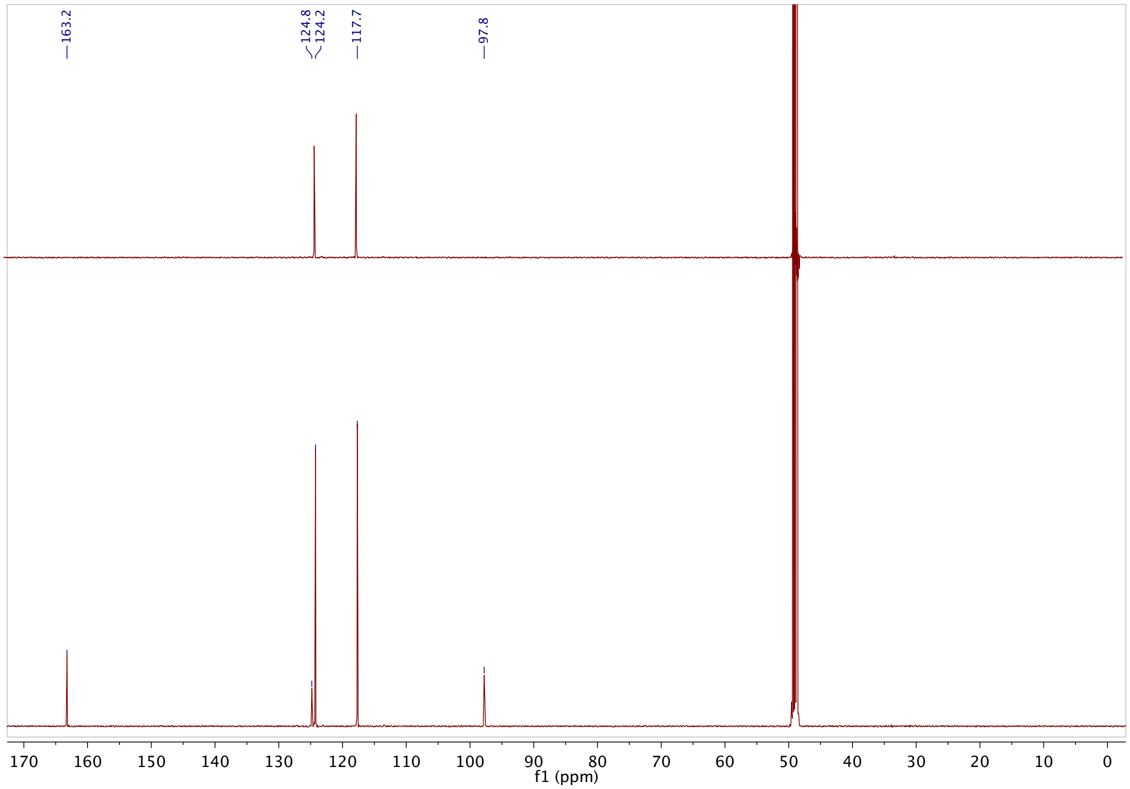

Figure S17. ^{13}C NMR and DEPT-135 spectra of compound **6** (125 MHz, CD_3OD).

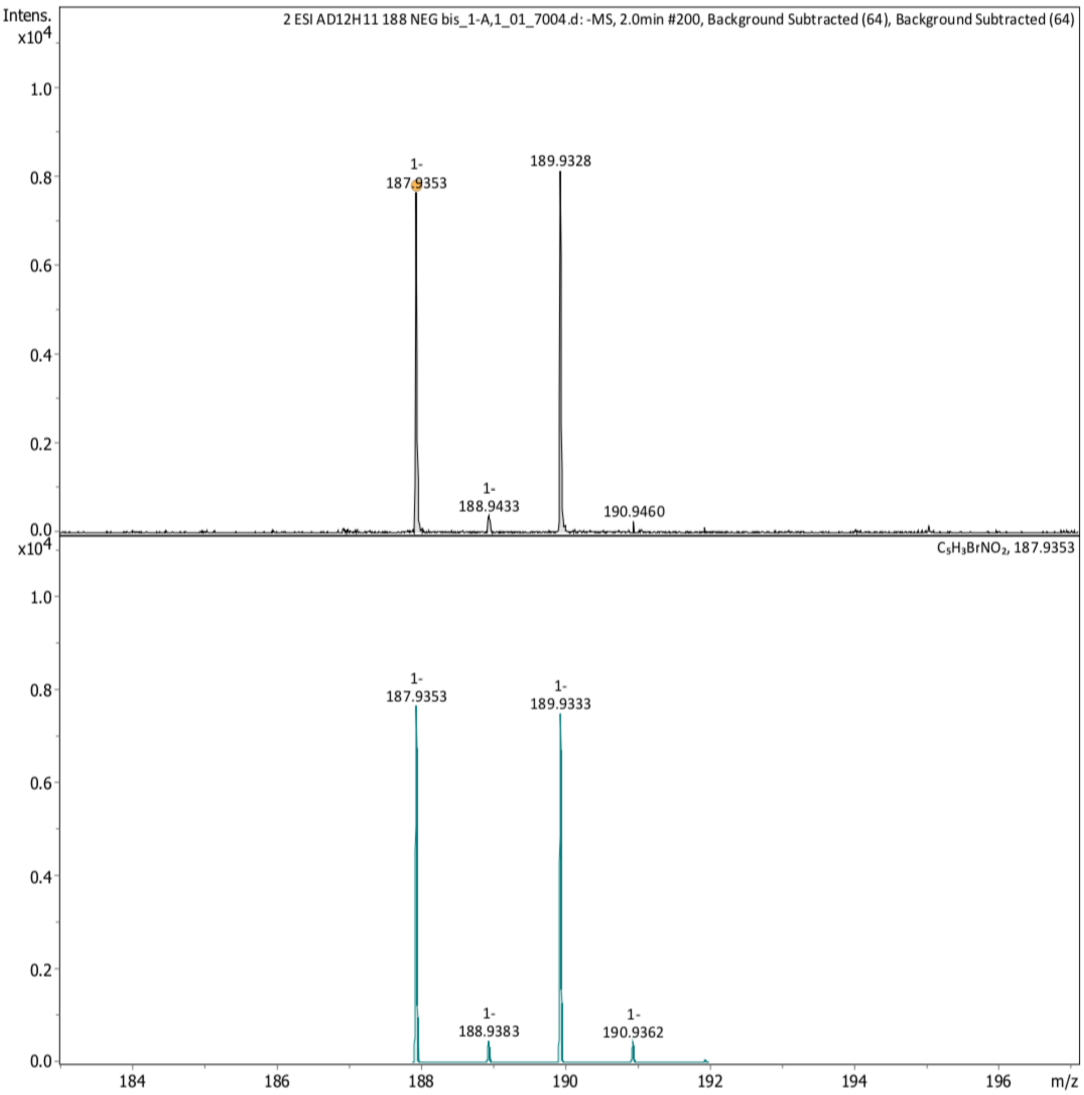

Meas. m/z	#	Ion Formula	Score	m/z	err [mDa]	err [ppm]	mSigma	rdb	e⁻ Conf	N-Rule
187.9353	1	C5H3BrNO2	100.00	187.9353	-0.0	-0.3	34.9	6.0	even	ok

Figure S18. (-)-HRESIMS of **6**.

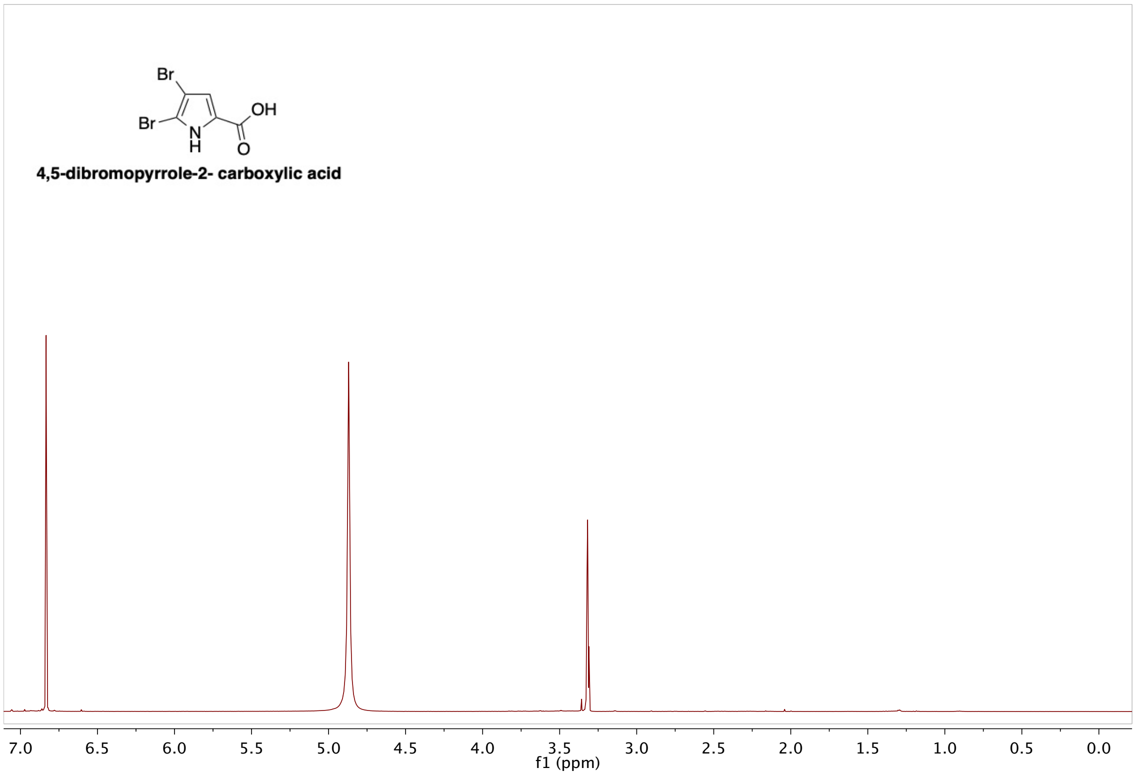

Figure S19. ^{1}H NMR spectrum of **7** (500 MHz, CD_3OD).

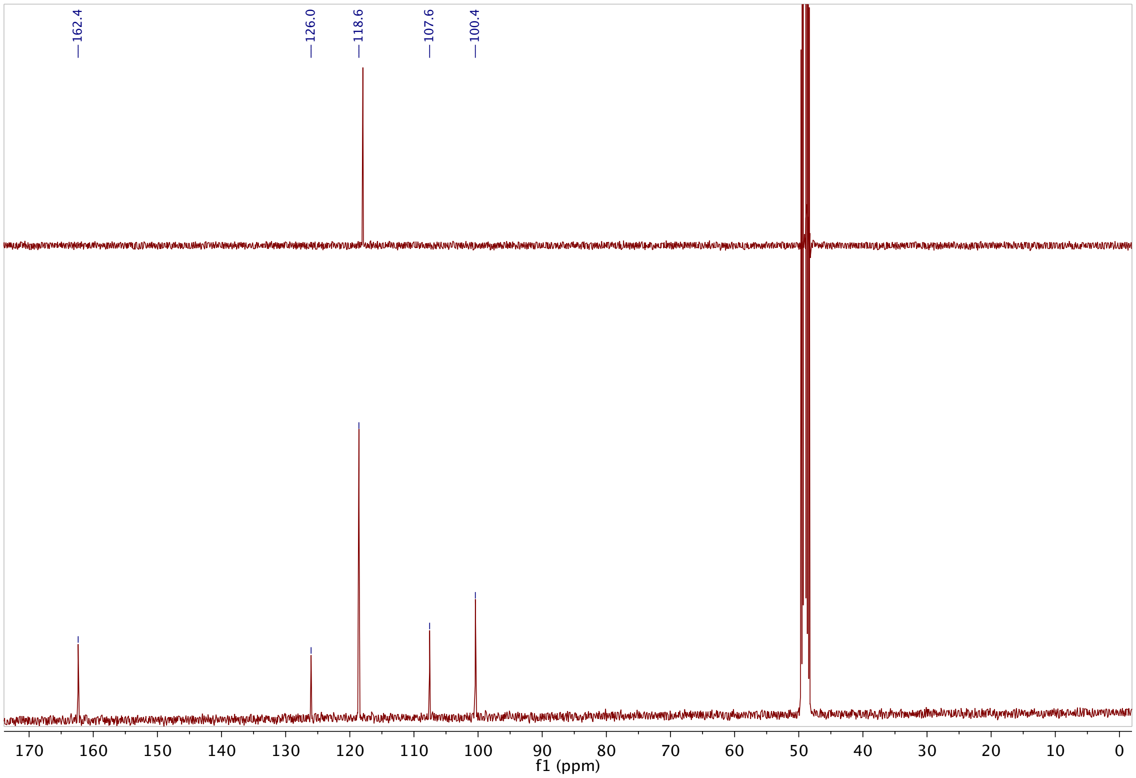

Figure S20. ^{13}C NMR and DEPT-135 spectra of **7** (125 MHz, CD_3OD).

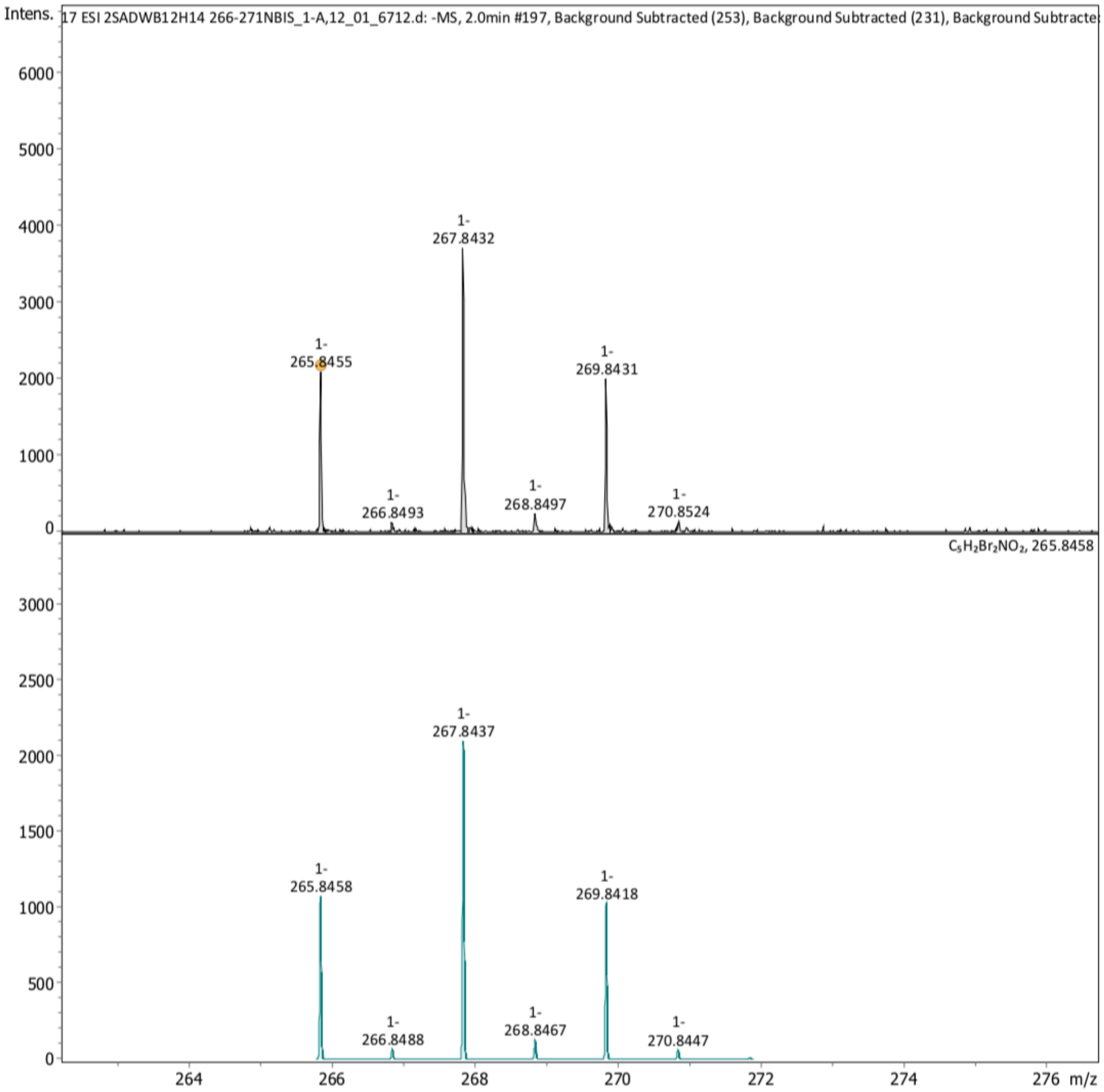

Meas. m/z	#	Ion Formula	Score	m/z	err [mDa]	err [ppm]	mSigma	rdb	e⁻ Conf	N-Rule
265.8455	1	C5H2Br2NO2	100.00	265.8458	0.2	0.9	28.7	8.0	even	ok

Figure S21. (-)-HRESIMS of **7**.

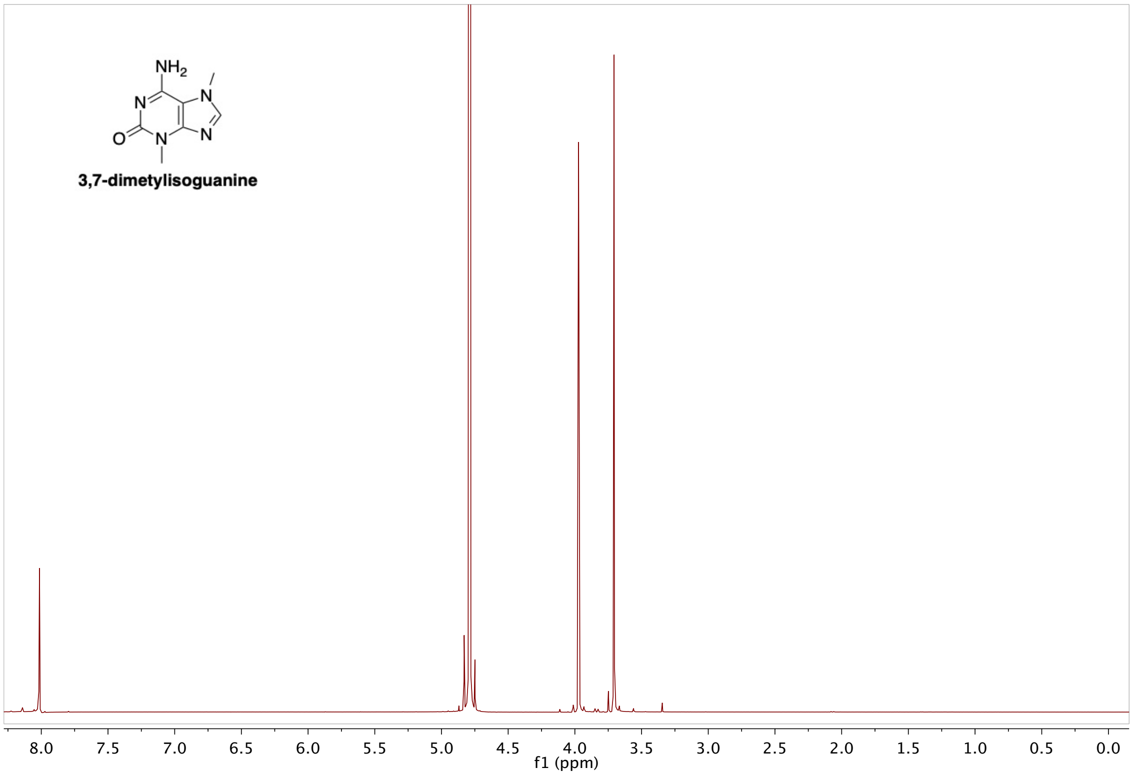

Figure S22. ^{1}H NMR spectrum of **8** (500 MHz, D_2O).

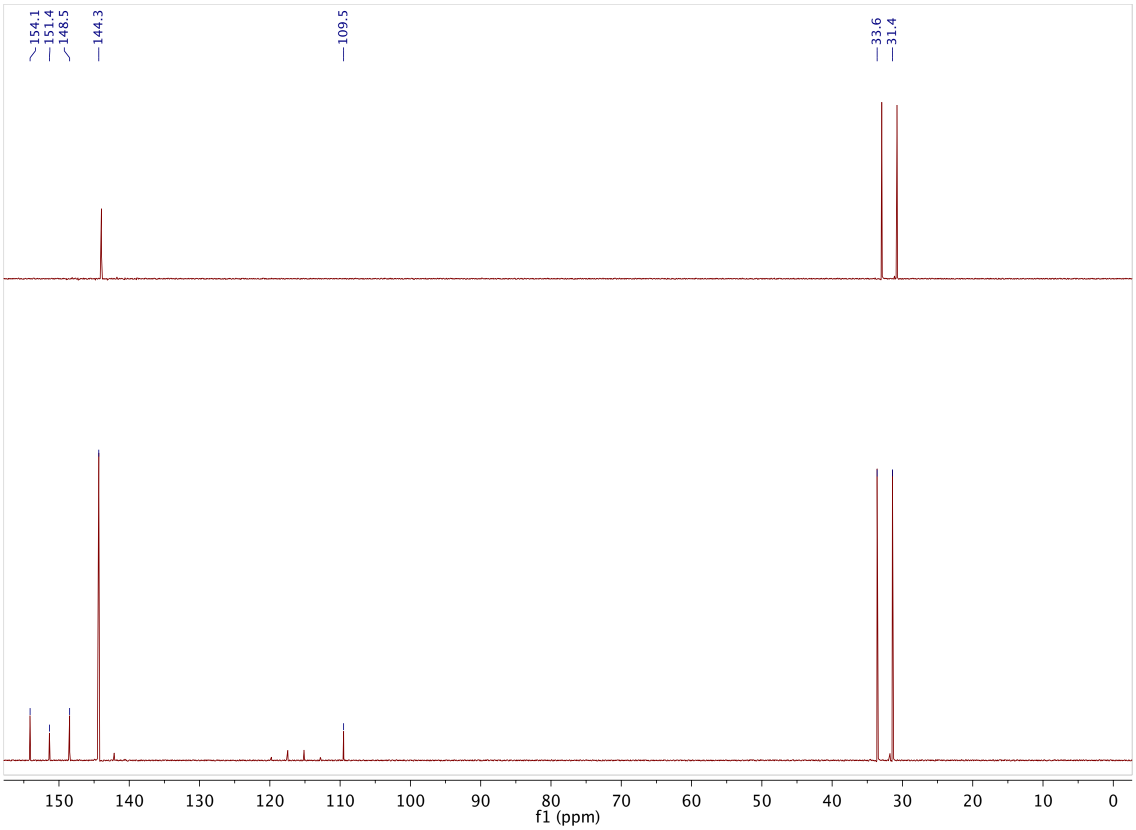

Figure S23. ^{13}C NMR and DEPT-135 spectra of **8** (125 MHz, D_2O).

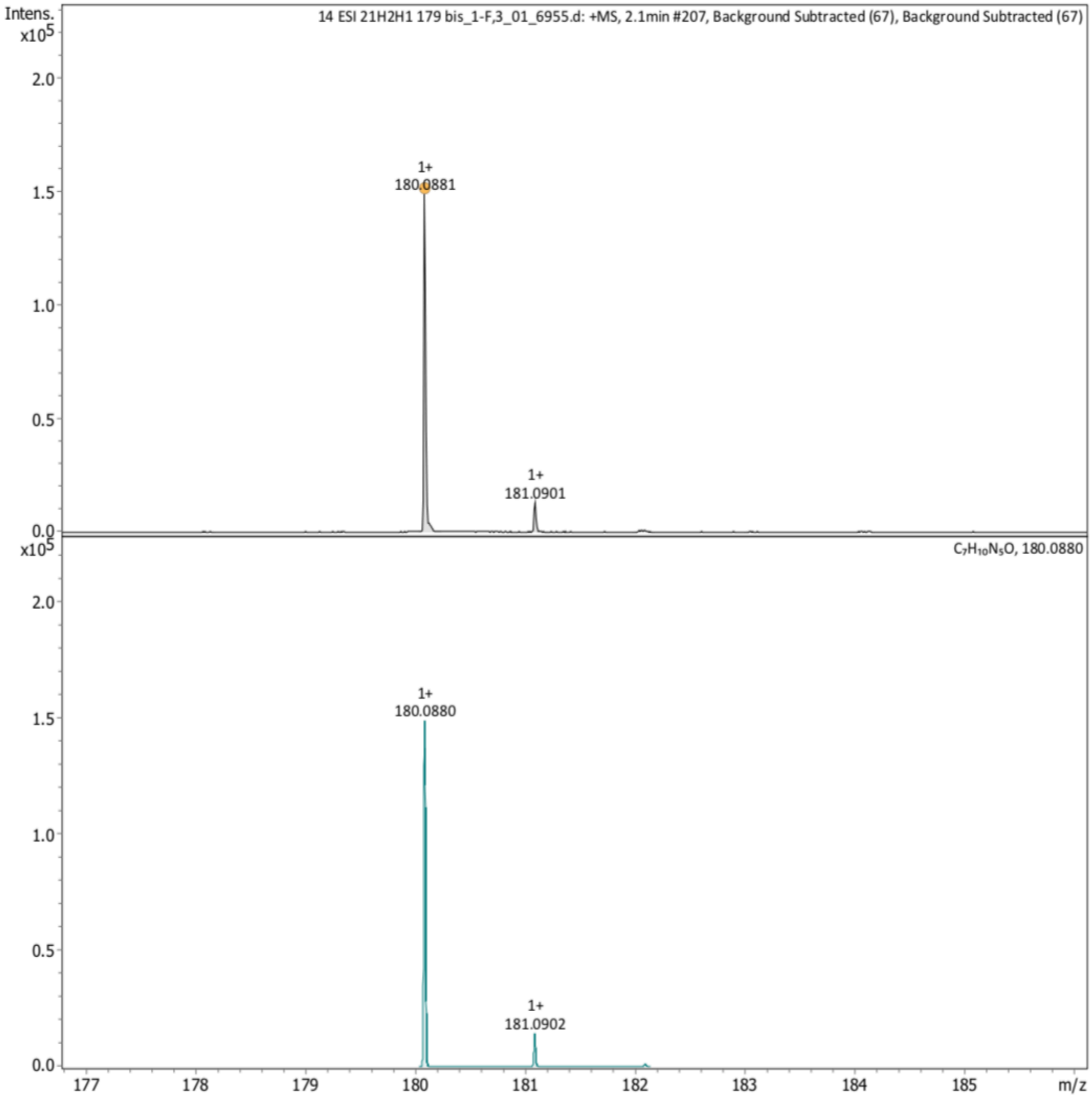

Meas. m/z	#	Ion Formula	Score	m/z	err [mDa]	err [ppm]	mSigma	rdb	e¯ Conf	N-Rule
180.0881	1	C7H10N5O	100.00	180.0880	-0.1	-0.7	6.1	6.0	even	ok

Figure S24. (+)-HRESIMS of **8**.
